# Recent Advances on Peptide-Based Biosensors and Electronic Noses for Foodborne Pathogen Detection

**DOI:** 10.3390/bios13020258

**Published:** 2023-02-11

**Authors:** Vanessa Escobar, Natale Scaramozzino, Jasmina Vidic, Arnaud Buhot, Raphaël Mathey, Carole Chaix, Yanxia Hou

**Affiliations:** 1 Grenoble Alpes University, CEA, CNRS, IRIG-SyMMES, 17 Rue des Martyrs, 38000 Grenoble, France; 2 Grenoble Alpes University, CNRS, LIPhy, 38000 Grenoble, France; 3 INRAE, AgroParisTech, Micalis Institute, Université Paris-Saclay, 78350 Jouy-en-Josas, France; 4 Institute of Analytical Sciences, University of Lyon, CNRS, Claude Bernard Lyon 1 University, UMR 5280, 69100 Villeurbanne, France

**Keywords:** peptides, antimicrobial peptides, phage display, biosensors, electronic nose, foodborne pathogen, pathogenic detection

## Abstract

Foodborne pathogens present a serious issue around the world due to the remarkably high number of illnesses they cause every year. In an effort to narrow the gap between monitoring needs and currently implemented classical detection methodologies, the last decades have seen an increased development of highly accurate and reliable biosensors. Peptides as recognition biomolecules have been explored to develop biosensors that combine simple sample preparation and enhanced detection of bacterial pathogens in food. This review first focuses on the selection strategies for the design and screening of sensitive peptide bioreceptors, such as the isolation of natural antimicrobial peptides (AMPs) from living organisms, the screening of peptides by phage display and the use of in silico tools. Subsequently, an overview on the state-of-the-art techniques in the development of peptide-based biosensors for foodborne pathogen detection based on various transduction systems was given. Additionally, limitations in classical detection strategies have led to the development of innovative approaches for food monitoring, such as electronic noses, as promising alternatives. The use of peptide receptors in electronic noses is a growing field and the recent advances of such systems for foodborne pathogen detection are presented. All these biosensors and electronic noses are promising alternatives for the pathogen detection with high sensitivity, low cost and rapid response, and some of them are potential portable devices for on-site analyses.

## 1. Introduction

A small group of less than ten microbes is responsible for causing humans millions of diseases around the globe every year. We ingest these foodborne pathogens when consuming contaminated water and food of all kinds, including seafood, poultry, dairy, fruits and vegetables. For most healthy adults, foodborne illnesses are not life threatening. However, complications may arise and result in serious conditions, such as septicemia, spontaneous abortion, stillbirth and death [1]. The World Health Organization (WHO) estimates that foodborne illnesses affect 600 million people and cause almost half a million deaths around the world every year, with children under 5 years old accounting for around 40% of them [2]. The most vulnerable populations are young children, pregnant women, immunocompromised patients and the elderly. Severe forms of these diseases may also occur due to the antibiotic resistance of pathogenic microorganisms, a problem mainly caused by the overexploitation of antibiotics in the medical, agriculture and food industries [3].

Foodborne pathogen contamination pathways include the contact of foodstuffs with water, sewage, air and soil during harvesting, processing and packaging. Bacteria, viruses, molds, worms, parasites and prions can be foodborne pathogens. Bacteria, however, cause the highest number of foodborne illnesses by far [4]. Although outbreaks vary regionally and affect countries of all incomes, least developed and developing countries are the most vulnerable. Africa, the Americas and the Eastern Mediterranean Region suffer the highest number of infections due to foodborne diseases per population mostly due to *Campylobacter*, *Salmonella*, *Taenia solium* and norovirus [2]. As for developed countries, in 2020, the European Food Safety Authority (EFSA) reported 3086 foodborne outbreaks, mostly being campylobacteriosis, salmonellosis and norovirus infections [5]. In Europe, annual monitoring is compulsory for eight zoonotic agents, of which *Salmonella*, *Campylobacter*, *Listeria* and Shiga toxin-producing *Escherichia coli* lead as the top pathogens [5]. Similarly, the U.S. identified *Salmonella*, Toxoplasma, *Listeria*, norovirus and *Campylobacter* as the top five foodborne pathogens in 2018 [6].

It is quite surprising that such a small set of pathogenic microorganisms could be responsible for millions of diseases worldwide. A factor that contributes to this annual reoccurrence is the inadequate reporting of outbreaks and their causes. For this reason, the WHO has emphasized the importance of identifying the most common foodborne pathogens by region, so as to generate targeted actions by regulatory bodies in the food industry [2]. Table 1 summarizes the classification and characteristics of the world’s top foodborne pathogens, current detection methodologies and regulatory limits in foodstuffs for the European Union.

Effective monitoring systems allow for their earlier detection, which prevents the loss of human life and lowers these diseases’ economic burdens, including costs of medical care, lost productivity and premature death related to foodborne illnesses. The U.S. alone estimated a 15.5 billion USD economic burden for the year 2018, highlighting that preventing foodborne diseases has become more economically valuable relative to other goods and services [6].

However, there continues to be an important gap between industrial needs, regulatory policies and existing detection technology. This disparity can be exemplified with the European Union’s mandatory screening of broiler carcasses for *Campylobacter* spp. since 2017, which has yet to be implemented, as a methodology that can fulfill detection demands does not yet exist. Another example is norovirus detection in the U.S., for which only one test, the RIDASCREEN Norovirus enzyme-linked immunosorbent assay (ELISA), is currently approved for diagnosis, but its use is authorized exclusively for outbreak settings due to its lack of sensitivity [7]. Thus, strategies for quality control improvement in foodstuffs must not be limited to the establishment of more strict measures and rigorous monitoring but should also take into account the development of detection systems that can reach the limits of detection needed in the industry through high sensitivity, cost-effectiveness and the feasibility of implementation.

The gold standard for foodborne pathogen detection is based on culture isolation in selective media coupled with serotyping, immunoassays or molecular biology methodologies for the identification of the specific species or strains [1]. Usually, the preliminary results are based on culturing on selective media, which is composed of the necessary nutrients for bacterial growth, as well as additional selective agents with the purpose of isolating a particular species or genus. This process takes 48 to 72 h, and in some cases, may require a pre-enrichment step of up to 48 h. Some bacteria, such as *Salmonella*, require serotyping, in which bacterial isolates are presented to antisera to identify characteristic antigens of different *Salmonella* serovars. However, this requires more than 150 specific antisera and highly trained microbiologists to interpret the results [8]. On the contrary, immunoassays take advantage of antibody–antigen specific interactions to measure the concentration of the pathogen in the sample. Although highly sensitive, these are not able to discriminate between live and dead bacteria, may be prone to false positives and negatives and are susceptible to cross-reactivity [9]. Molecular biology techniques, on the other hand, focus on the recognition and exponential amplification of short nucleic acid fragments specific to a target. One of the most widely used techniques is polymerase chain reaction (PCR), which is compatible with multiplex detection by the use of additional specific primers. Although highly sensitive and specific, this technique is susceptible to inhibitors [10] and needs specialized equipment. Other DNA amplification strategies performed at a constant temperature have been developed to circumvent the use of a thermocycler, such as loop-mediated isothermal amplification (LAMP). However, this technique is extremely sensitive and thus susceptible to contamination that could lead to false-positive results [11].

Although most classical methodologies are accurate and reliable, they can be expensive and require specialized equipment and personnel. Furthermore, the current monitoring processes are lengthy, requiring up to one week for species confirmation. Thus, their implementation time frame is not compatible with the preventive approach that legislative regulation often aims for.

**Figure 1:**
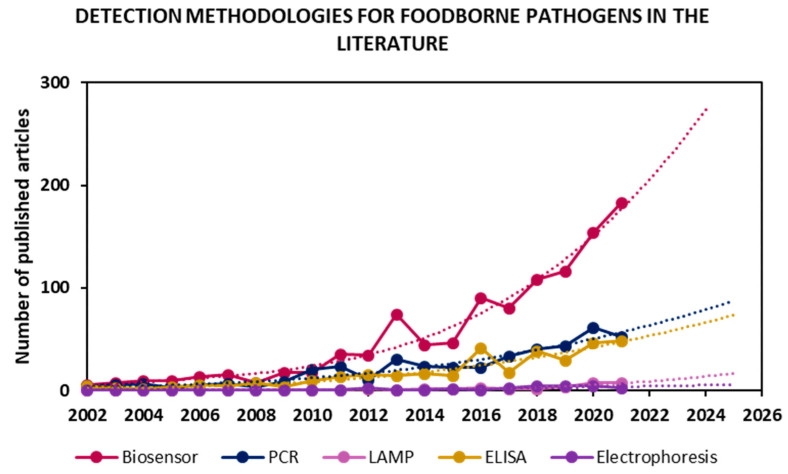
Detection methodologies for foodborne pathogen detection in the literature in the period of 2002–2021. Values were obtained by searching “foodborne pathogen detection” and synonyms as keywords in Scopus, then classifying 2424 articles found in the literature by methodologies. Trends obtained by fitting a tendency curve and projecting it for the next 4 years.

In an effort to address these shortcomings, recent years have seen a clear peak in the development of systems for the detection, discrimination and identification of pathogenic microorganisms in a rapid manner and in accordance with regulations. Figure 1 shows articles published for “foodborne pathogen detection” of the most used sensing methodologies, along with future trends according to their publication rate in the last twenty years.

Many works have focused on the improvement of already implemented approaches, such as PCR and ELISA, while emerging technologies such as LAMP have only recently gained interest. However, the fastest-growing research field is biosensors. Furthermore, the rate of publication in each field over the last 20 years was projected onto the next four years, and, once again, it seems that the biosensor field’s exponential growth will dominate pathogen detection research. The main objectives in the biosensing field are the development of highly sensitive, low-cost, rapid, portable devices that are compatible for on-site testing and have the same or better performance than the currently implemented techniques. Indeed, the WHO has published international guidelines for new diagnostic tools known as REASSURED (Real-time connectivity; Ease of specimen collection; Affordability; Sensitivity; Specificity; User-friendliness; Rapid & robust operation; Equipment-free; and Deliverability) [12].

**Figure 2:**
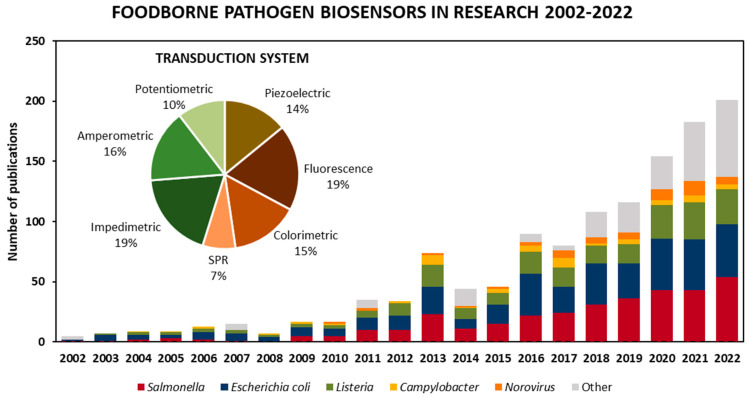
Published articles on biosensors for the detection of foodborne pathogens in the literature in the period of 2002–2022. Values obtained by limiting “foodborne pathogen detection” and synonyms as a keywords article search in Scopus from 2002 to 2022 to biosensors, then classifying the 1167 articles by target foodborne pathogen and transduction techniques.

As for the targeted microorganism, Figure 2 shows published articles in biosensors for foodborne pathogen detection according to the targeted microorganism from 2002 to 2022, as well as the total percentage of the transduction technique employed. Various types of biosensors have been developed by focusing on the detection of *Escherichia coli* and *Salmonella*, as they represent a heavy burden to the clinical and food safety domains. Nevertheless, there has been a disproportionate focus on the detection of other pathogens, such as norovirus, *Campylobacter* and *Listeria*, which have been responsible for an even larger number of illnesses in recent years and, in some cases, are more likely to be deadly [5]. However, Figure 2 shows that the food safety field continues to expand as research steers towards targeting a wider range of foodborne pathogens, such as *Staphylococcus*, *Pseudomonas aeruginosa* and *Bacillus*, included in the “other” category.

Among different types of transduction systems used, electrochemical (impedimetric, amperometric and potentiometric) biosensors are the most widely employed, making up almost 45% of the biosensors found in the literature search. Next up, optical biosensors, including fluorescence, colorimetric and surface plasmon resonance (SPR)-based platforms, make up about 41%, and the remaining 14% are mass-based biosensors such as quartz crystal microbalance (QCM), surface acoustic wave (SAW) and nanomechanical systems.

Different bioreceptors were used to construct these biosensors, including antibodies [13,14], nucleic acids [15,16], aptamers [17,18], peptides [19,20,21,22] and bacteriophages [23,24]. The choice of bioreceptor is paramount to achieve reliable detection with high sensitivity and specificity.

Antibodies were the logical first choice for the development of sensitive detection instruments, having extremely high specificity and affinity for their target [25]. However, their production is laborious, time-consuming and expensive. Additionally, they are more sensitive to unfavorable environmental conditions and can lose stability and specificity for their target under certain complex conditions.

Nucleic acids, such as DNA and RNA, have been explored due to their ability to recognize microorganisms based on a specific genetic sequence. As a notable example, real-time PCR (RT-PCR) has quantitative and qualitative characteristics and has been previously used to evaluate various foodborne pathogens [26]. The main drawbacks of nucleic acid-based methodologies are the use of specialized equipment or personnel, high cost, most of these techniques’ incompatibility with on-site detection or to distinguish between live and dead bacteria and their dependence on the DNA polymerase enzyme. Indeed, even the presence of low amounts of ions and molecules from food matrices in extracted nucleic acids may inhibit DNA polymerase and prevent amplification.

Aptamers are short, single-stranded nucleic acid sequences that have high binding affinities to different targets and are able to adopt specific three-dimensional sequence-dependent conformations. They are identified and selected through the systematic evolution of ligands by exponential enrichment (SELEX) methodology according to their ability to bind to a specific target [27,28]. After undergoing three-dimensional folding, a binding site is created. Aptamer–target interactions are dependent on the complementarity of their shapes, rather than the genetic sequence. Nevertheless, this binding event is capable of reaching levels of specificity comparable to those of antibodies. Some of their advantages include their stability, easy synthesis, high yield of production and the possibility to form multiple combinations of nucleic base “building blocks”, which allows for the creation of multiple candidates that can be screened against a target [29].

Peptides have gained interest in the biosensing field thanks to their unique features, such as good biocompatibility, high stability, ease of synthesis and sequence versatility. Indeed, compared to antibodies, peptides are more resistant to harsh conditions, such as high temperatures or wide pH ranges, required for on-field applications [20]. Today, there are various biological and chemical techniques for the rapid screening of peptide libraries, and their synthesis is simpler and cost lower compared to other biomolecules used in biosensors, such as antibodies or nucleic acids [30]. Furthermore, natural and synthetic peptides may contain D-amino acids, which are enantiomers of L-amino acids and have been considered as non-natural amino acids for a long time [31]. Interestingly, almost all bacteria contain D-amino acids, such as D-alanine and D-glutamate, in their cell envelopes. Peptides carrying D-amino acids bind efficiently to bacterial cells through the incorporation of their D-amino acids into the bacterial cell wall, as demonstrated for *Bacillus subtilis* [32].

Besides the aforementioned biomolecules, bacteriophages (phages) have also been used as bioreceptors. Phages are viruses that specifically recognize their bacterial hosts in order to infect them and replicate. They may be lytic or nonlytic, depending on whether they lyse the bacterial membrane after replication or not, and may be used for bacterial quantification assays, done by measuring the adenosine triphosphate (ATP) concentration through bioluminescence or other bacteria cytoplasmic markers that are quantifiable after membrane disruption. Furthermore, phages can be easily implemented in electrochemical sensors, as the disruption of the membrane upon bacterial binding causes a drop in conductivity [33], an easily measurable event. The production of phage clones with identical genetic sequences is easy and inexpensive, as host infection results in their replication into thousands of copies. Additionally, phage probes can differentiate between live and dead bacteria, withstand harsh conditions, such as wide ranges of pH and temperature, and may also be used as signal amplifiers. Every step of the phage infection cycle has been exploited for detection techniques, but only a few of these have been developed into commercial products, as they have yet to prove a significant advantage in rapidity, sensitivity or specificity over existing techniques [34]. Although the use of phage clones ensures better repeatability due to the robustness of the particle itself, the effective immobilization of whole bacteriophages onto substrates is a crucial step that might prove difficult, as there are multiple possibilities with differing optimal conditions, depending on the orientation, the surface and the type of immobilization chosen [35].

So far, in the literature, the use of antibodies and aptamers in the foodborne pathogen detection field has been extensively reviewed [36,37,38,39], in contrast to peptide-based biosensors. Therefore, in this review, we will focus on the sensitive and selective biosensors developed using peptides as bioreceptors for the detection of the most prevalent foodborne pathogens. Moreover, we will present an overview on other emerging peptide-based sensor techniques, such as electronic noses (eN), for foodborne pathogen detection.

## 2. Strategies for Peptide Selection

Peptides are chains of covalently linked amino acids. There are 20 natural L-amino acids, all consisting of the same framework and differing side functional groups that confer them different physicochemical characteristics. Upon interactions with each other, they may acquire a specific spatial conformation. A peptide containing *n* amino acids may be arranged in 20*^n^* possible ways. Thus, the combination of this relatively small set of different building blocks results in an enormous diversity in structure and biological activity. This seemingly endless possibility of combinations makes peptides particularly attractive as bioreceptors. Through designing an amino acid sequence, one is able to obtain whatever physicochemical and structural characteristics are required for the detection of a given target or for a specific application. Hydrophobicity, polarity, length and even rigidity can be modulated quite easily by adjusting its amino acid constituents, as well as the enhancement of its selectivity and specificity to a given target [40].

**Figure 3:**
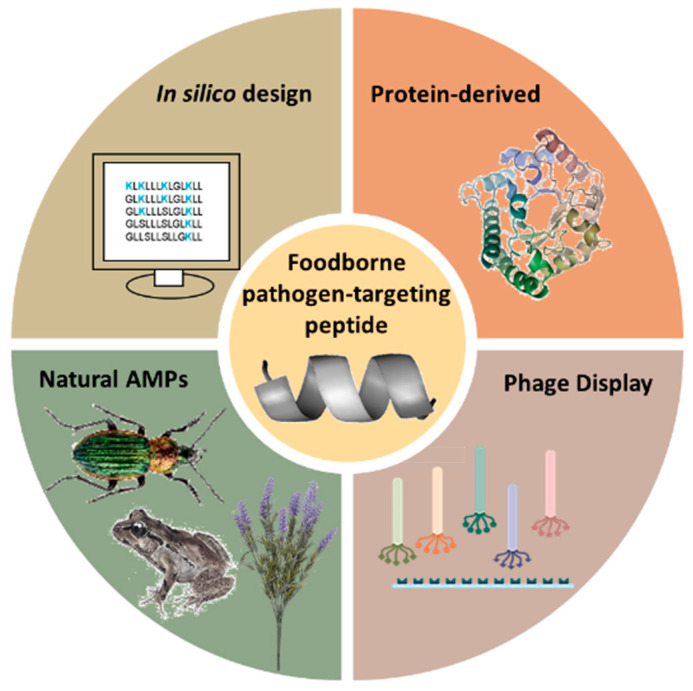
Different strategies for the design of foodborne pathogen-targeting peptides.

The choice of amino acid sequence in peptides for biosensor probes is anything but arbitrary. Numerous methodologies have been developed to find the precise conformation that will result in the required selectivity and specificity towards a target—in this case, a foodborne pathogen. Some of the most notable peptide selection strategies, shown in Figure 3, are the isolation and purification of natural antimicrobial peptides (AMPs) from living organisms, screening short-peptide libraries using genetically engineered bacteriophages with a phage display, rational in silico designs and protein-derived approaches.

### 2.1. Antimicrobial Peptides

Antimicrobial peptides are naturally occurring molecules present in virtually all living organisms as a line of defense against the various pathogenic microbes to which we are constantly exposed. Contrary to the mechanism of action of antibodies comprising the adaptive immune system, AMPs target microbes without specificity. This broad-spectrum antimicrobial activity is accomplished by targeting the negatively charged motives in the bacterial envelope, such as the phospholipid head groups of bacterial membranes, or some oligosaccharides in the cell wall, absent in eukaryotes [41].

AMPs are particularly important to those organisms that cannot afford the biological cost of having an adaptive immune system, such as insects, invertebrates and even bacteria themselves. A nomenclature for AMPs has not been standardized yet, so their classification is mainly based on their organism of origin noted with the –cin suffix [42], i.e., human defensins, bactericins, etc.

The first records of antimicrobial activity identified in a substance derived from a living organism date back to the 1920s, with Alexander Fleming’s discovery of bacterial lysis upon contact with nasal secretions from patients. He named the protein responsible for this phenomenon “lysozyme” [43]. Further interest in antimicrobial substances arose in the 1980s, when three peptides identified and purified from the giant silk moth *Hyalophora cecropia* were shown to have bacteriolytic activity against *Escherichia coli* [44]. Ever since, a plethora of unique AMPs have been identified from all sorts of living organisms, such as Magainin I, originally isolated from the skin of the African clawed frog *Xenopus laevis* in 1987 [45], or Clavanin A, purified from the tunicate *Styela clava* [46]. To this day, more than a thousand different AMPs have been identified, and a partial list can be found in the database APD3 (https://aps.unmc.edu, accessed on 30 December 2022) [42].

Although the exact way in which these molecules disrupt the bacterial membrane remains unknown, a few hypotheses have been formulated regarding AMPs’ mechanism of action. These include adsorption to proteins and lipids from the membrane surface, nonlytic depolarization, solubilization of the membrane into micellar structures [47], the disruption of the osmotic regulation of the target bacteria [48] and the ability to hijack biological processes crucial to bacterial survival, such as DNA and protein synthesis.

Amongst the vast diversity of isolated AMPs, a common physicochemical feature stands out: an amphipathic conformation consisting of a cationic polar portion and a hydrophobic domain. This duality permits an initial interaction of the net positive charge fragment of the peptide with the negatively charged bacterial membrane, followed by the insertion of the peptide into the membrane, mediated by hydrophobic interactions [49]. It has been hypothesized that the peptide’s secondary structure plays an important role in this process, as helical structures and beta sheets may be able to present a continuous hydrophobic surface, advantageous for peptide–bacteria interactions.

This mechanism of action is instrumental to the fact that bacterial adaptive resistance to AMPs is rare, as, to circumvent AMPs, bacteria would have to modify their membrane, which constitutes a large proportion of their total composition. However, when the targets are small proteins within the cell, which is often the case for antibiotics, this genetic modification might be much easier to perform, resulting in an easier acquired resistance [41]. For this reason, AMPs have been explored as alternatives to antibiotic treatment, especially when dealing with multidrug resistance organisms [50].

AMPs lack the specificity of monoclonal antibodies but are nonetheless exceptional at recognizing and selectively interacting with bacteria [51]. Further selectivity is achieved by targeting specific lipopolysaccharide (LPS) compositions, as they are highly variable between genera, species and even strains, differing by the number and structure of repeating oligosaccharide units [52]. In the biosensor field, AMPs’ binding capabilities make them excellent probe candidates for the development of highly sensitive multiplexed arrays, especially those in which the main priority is the confirmation of total sterility or the presence of pathogenic bacteria, rather than the identification of a specific species. Table 2 includes a list of AMPs that have been incorporated into biosensors for the detection of foodborne pathogens. Other areas of application of AMPs in the food industry, such as food preservation, the development of antimicrobial packaging and the formulation of antibiofilm sanitizing products, have been extensively investigated in outstanding reviews [53,54,55].

Synthetic antimicrobial peptides have also been thoroughly explored in recent years, mostly with a focus on clinical treatments due to their ability to kill antibiotic-resistant pathogens and because they rarely trigger resistance mechanisms in microorganisms [66]. Although the mechanism of action of AMPs has not yet been fully elucidated, novel antimicrobial peptides can be designed based on their known defining characteristics, such as their short length, the formation of helices and β-sheets as secondary structures, their cationic net charge and their amphipathic nature.

In the literature, some research groups improved specific peptides for pathogen detection by rational design. To do so, first, AMP sequences were isolated from living organisms, which generally resulted in the elucidation of the structure and design requirements for the synthetic construction of novel AMPs. Then, small mutations, such as deletions of a single amino acid, were made to increase their net charge, improving their selectivity towards bacterial pathogens [67].

Rational design may also be used to confer specificity to an existing partially selective antimicrobial peptide through the addition of a species-specific targeting domain to a broad-spectrum AMP. For example, Eckert et al. screened a set of short (8 to 12 amino acids) fluorescently labeled peptides with varying physicochemical characteristics against *Pseudomonas* spp. to find the strongest binding candidates. Out of those, a single peptide exhibiting selectivity against *Pseudomonas* (KKHRKHRKHRKH) was identified. They then introduced a linker sequence (GGSGGS) to incorporate the selective short peptide onto the C-terminal of Novispirin (KNLRRIIRKGIHIIKKYG), an AMP known to have lytic activity against a wide range of bacteria. The resulting chimeric peptide was able to selectively retard *Pseudomonas* growth and leave *E. coli* and *Streptococcus mutans* virtually unaffected [68].

### 2.2. Peptides Screened by Phage Display

Phage display is a Nobel Prize-winning in vitro technique that selects phage clones expressing the peptides with the highest affinity to a specific target from an initial pool of candidates [69]. The process closely resembles evolution, as it relies on the fabrication of billions of “candidates” through DNA recombination in viral particles and the selection of the fittest through consecutive cycles of high-affinity purification. The application of phage-displayed probes for biosensors in various fields has been recently discussed in several reviews [70,71].

In order to find a peptide with high affinity to a selected target, an initial “library” is generated by inserting random foreign DNA sequences into bacteriophages, which, in turn, express the corresponding peptide on their outside coating. This will result in a heterogeneous mix of around a billion phage clones bearing different foreign peptides. The most commonly used type of phage for this technique is the M13 bacteriophage, which possesses five different coat proteins: pIII, pVI, pVII and pXI present in five copies each, and pVIII presents in around 2700 copies all along its filament [72]. In general, phages express foreign peptides with between 7 and 15 amino acids, depending on the library.

Phages expressing foreign peptides on the pVIII coat protein, such as those from the f8/8 library, express thousands of foreign peptides in a compact, reiterating pattern over the whole length of the phage capsid [73]. These are also called “landscape phages”, as this multivalent display of the foreign peptide may result in the activity of not only the single peptide and its immediate surroundings but also global functions of the entire surface “landscape” [74]. By contrast, a monovalent peptide display refers to its expression solely on pIII, present in five copies at one end of the filament. Monovalent display libraries are the most commonly used for bacterial targets.

The classical phage display protocol consists of six main steps [75]: First, the incubation of the initial phage library takes place over a surface on which the targets have been previously immobilized. Upon incubation, the phages with higher affinity to the target are attached, and those with low or no affinity remain unbound in the solution. The subsequent washing and elution steps with increasing levels of stringency apply selective pressure, the former to remove unbound phages and the latter to release bound phages with a higher affinity for the target analyte. Both steps must be performed without losing the virion’s infectivity for the next step to take place. Afterwards, *E. coli* host cells are added, and the process of amplification begins as the phages infect host cells for replication and, consequently, thousands more copies of those phages expressing the fittest peptides are produced. Finally, after three or four selection cycles (steps 1–4), the amino acid sequences of the few selected peptides with the highest affinity for the target are determined by sequencing the genetic material of the phage. They can then be synthesized and used as bioreceptors for the development of biosensors.

Phage displayed peptides have been used for foodborne pathogen detection because of their remarkable selectivity towards a specific species or strain, contrary to the wide-spectrum binding of other peptides. Table 3 shows a list of peptides selected by phage display with a foodborne pathogen as the target.

The phage display approach yields the consensus sequences of the best binding candidates under a specific set of experimental conditions. However, different sequences may be found upon making slight changes [69]. Thus, there are a few challenging aspects to take into account for a successful experimental design.

For example, bacterial membranes are complex mixtures of proteins and phospholipids and express different antigens on their surfaces [89], which makes experiments more challenging than when dealing with simpler and smaller targets. Furthermore, the surface epitopes bacteria expressed when immobilized may be different to those expressed in the solution and different still to those expressed when in natural environments or food matrices. In this case, it is not surprising that the peptides obtained from immobilized bacteria would be different to those found when that same target is suspended in a buffer solution. However, a conclusion has not been reached on whether one strategy is inherently better than the other. Sorokulova et al. compared various methodologies when biopanning the f8/8 phage against the target *Salmonella enterica* Typhimurium, presenting it both in the solution and surface-immobilized for comparison. They found the peptide with the highest specificity when using immobilized bacteria [80]. On the contrary, McIvor et al. tested a comparison between the two methodologies and found the opposite: for *L. monocytogenes*, the clones with specific binding to the serovar were found exclusively when working with bacterial suspensions in Phosphate-Buffered Saline (PBS) buffer [88].

During a phage display experiment, non-target-specific peptides may be enriched inadvertently [90]. Peptides with specificity to either the immobilization surface [91], the blocking solution, the capture molecule (biotin or streptavidin) or support of the target are called “target-unrelated peptides” (TUPs) [92]. In order to avoid them, “subtractive biopanning” is advisable—that is, incubating the initial library onto the biopanning infrastructure containing all elements except the target to eliminate all potential TUPs. The most comprehensive list to date of TUP amino acid sequences reported in the literature is the “Scanner And Reporter Of Target-Unrelated Peptides” (SAROTUP) [93,94]. Subtractive biopanning may also be used to find a peptide with higher selectivity. In this case, negative selection can be done by incubating the library with another microorganism similar to the target in a Gram stain or species in order to deplete it of those clones binding to motifs common to several bacteria and eliminating the possibility of cross-reactivity later on. Rao et al. used this strategy when biopanning against *Staphylococcus aureus*, performing pre-adsorptions against *E. coli* to subtract clones binding to Gram-negative bacteria and subsequently against *Staphylococcus epidermis* to eliminate those binding to the *Staphylococcus* genus [83]. In contrast, McIvor et al. opted to perform negative selection in the last biopanning round, incubating peptides with *Listeria innocua* from the same genus as the target, *L*. *monocytogenes* to find peptides with higher specificity towards the latter [88]. These data show that the biopanning step is crucial to obtain the most selective sequences, and there is not one experimental solution but a conjugation of selection steps that can help to obtain target-specific sequences.

Interestingly, it is precisely the limitations of a blind, unbiased experiment that can be used to our advantage. Phage display has recently gained interest for finding new specific receptors that could identify surface motifs or structures that may not have been previously identified by other approaches [79]. Additionally, an exceptional advantage of phage display is the fact that it bypasses the requirement for target immunogenicity, one of the main limitations of in vivo antibody production [95]. Furthermore, a bacterial strain may not express surface epitopes that are both unique and antigenic, which would render the produced antibodies incapable of discriminating between strains of the same genus [96]. McIvor et al. reported a notable example when testing commercial antibody-coated beads that were not able to differentiate between *L. monocytogenes* and *L. innocua*. They hypothesized that, in *Listeria*, the immunodominant epitopes may be shared between species, making it difficult for antibody probes to identify the less immunogenic but crucially specific epitopes that differentiate serovars [88].

Additionally, the accurate detection of highly mutagenic foodborne pathogens, such as Norovirus, would likely need new specific probes to be developed every few years. In this case, the ease of adaptability and low cost of the phage display methodology would make it a much more attractive approach than the cost and laboriousness of constant antibody development [7].

It is important to note that the categories of peptides mentioned in this review are not mutually exclusive, and some phage-displayed peptides may meet the same structural and net charge criteria as AMPs of living organisms, thus exhibiting antimicrobial activity against bacteria [79,97].

This review focuses specifically on biosensing platforms using peptides as detection probes; however, either the synthetized specific peptide or the entire phage may be incorporated in biosensors. The use of phages displaying specific peptides as sensing elements has been thoroughly reviewed in previously published reviews [98,99,100,101].

### 2.3. In Silico Design of Peptides

Another strategy for peptide rational design is based on in silico tools. Indeed, in the last two decades, molecular simulations have shown to be a powerful theoretical technique to study peptide structures and dynamics [102,103,104]. There are three main approaches for designing antimicrobial peptides: the modification of known AMP sequences, biophysical modeling and virtual screening [102].

First, the sequence modification approach consists of using known AMP sequences as templates and subsequently modifying of one or more amino acids to identify the most crucial amino acids and their positions for antimicrobial activity or to elucidate the role of certain motifs present in the peptide on its overall mechanism of action [105]. Wiradharma et al. designed short AMPs by using repeats of hydrophobic and cationic residues known to confer antimicrobial activity. In this way, they found peptides with increased antimicrobial activity against Gram-positive bacteria and selectivity towards microbial cells [106]. Likewise, chimeric peptides with increased selectivity can be designed by combining motifs of wide-spectrum AMPs with targeted ones [107]. Second, biophysical modeling relies on the design of AMPs based on structural motifs and their properties, accounting for their interactions with the bacterial membrane and the media around them. In this case, the use of molecular dynamics simulations can lead to the improvement of antimicrobial activity. Third, virtual screening approaches are used to explore sequence iterations that may prove too difficult to test using other screening techniques. These include the use of bioinformatics tools, such as machine learning methods, evolutionary algorithms and stochastic approaches. The development of online software capable of predicting AMPs derived from a given protein, such as the “Antibacterial peptides” (AntiBP) and the “Collection of Antimicrobial Peptides” (CAMP) servers, have facilitated the design of new peptides by using Quantitative Matrices, Artificial Neural Networks and Support Vector Machines. Recently, Yang et al. predicted, designed and validated an AMP derived from the sequence of the small subunit of *Penaeus vannamei* hemocyanin (PvHS) using these servers. Two out of the twelve predicted peptides showed strong antimicrobial activity on Gram-negative and Gram-positive bacteria [108]. Subsequently, the team synthetized them and performed a structural analysis revealing a β-sheet structure, and scanning electron microscopy confirmed the peptide’s ability to disrupt the bacterial membrane.

### 2.4. Protein-Derived Peptides

Furthermore, a library of short peptides may be produced and screened using larger proteins or enzymes with specific activity as the starting templates. For example, Palmieri et al. combined in silico predictions and docking simulations to design short peptides from the protein CPT-1A (carnitine palmitoyl transferase 1a), predicting advantageous mutations that would confer increased antimicrobial activity to candidate peptides. In this way, the team found two peptides with antimicrobial activity against *L. monocytogenes* [109]. In another recent example, Mardirossian et al. designed short peptide fragments from the larger 25 amino acid peptide Bac5, a proline-rich AMP, for their use as antibiotics and tested their activity against *E. coli*, *S. aureus*, *P. aeruginosa* and other bacteria. They found the minimum length required for mammalian AMPs to keep their antimicrobial activity is 17 amino acids [110].

## 3. Overview of Peptide-Based Biosensors for Foodborne Pathogen Detection

Once the peptide bioreceptors have been selected, the next critical step is to graft them onto a sensor surface for target detection. Peptides are well suited for biochip functionalization, as they are resistant to air-drying without a significant loss of activity [111]. Furthermore, they can be easily grafted with different surface chemistry strategies. Effective target binding can be largely dependent on the immobilization methodology, as it plays a significant role in the number of nonspecific binding events, the amount of background noise and the reproducibility and repeatability of biosensor manufacturing. Several strategies for peptide immobilization have been tested and compared in the quest to find the most efficient one [112]. Different sensor setups may have specific requirements. Furthermore, the orientation of the immobilized peptide on a gold surface can be easily chosen by adding the cysteine anchor at either the C- or the N-terminus. Several groups have found that the best binding and the highest antimicrobial activity are maintained when immobilizing peptides through the addition of cysteine at the C-terminus, suggesting that, in vivo, the free N-terminal performs the first interaction with the bacteria due to its positive charge [113,114,115].

Short linear peptides can reach a much higher surface density than other biomaterials and are able to form a uniform grafted layer due to the spontaneous formation of self-assembled monolayers (SAMs) onto gold surfaces. This is advantageous for biosensors, as it has been observed that the ability of a peptide to capture target bacteria is strongly dependent on its concentration and density on the sensor surface [112,114]. Additionally, bacterial binding to peptides results in a lower steric hindrance than to antibodies, allowing for a higher binding avidity for the target per surface unit [77]. To further increase the immobilization surface area, peptides may also be grafted onto nanoparticles. This is especially interesting when testing extremely small sample volumes that require a higher surface-to-volume ratio, which is the case for microfluidic devices [116]. In another recent work, Baek et al. tested the importance of the flexibility on peptide–target interactions by introducing a rigid linker (–EAAAK-) rather than a flexible one (–GGGGS-), both at the C-terminus of the peptide and in the middle, and comparing their performances to those of the native sequences. They found the best binding to Norovirus when introducing the flexible linker, highlighting the advantages of using these short molecules as flexible probes [40].

Peptides have been used in a wide variety of biosensors for foodborne pathogen detection, as seen in Figure 2. They target either the bacterial surface or molecules emitted from bacteria to the medium. Consequently, peptide-based biosensors do not require complex sample preparation prior to analysis, which significantly shortens the analysis time and enables rapid and low cost diagnostics in foodstuffs. The majority of the developed platforms have had different transducer methodologies, such as electrochemical, optical, mechanical or hybrids of the aforementioned. In the following section, an overview of peptide-based biosensors based on various transduction systems for foodborne pathogen detection is presented.

### 3.1. Electrochemical Peptide-Based Biosensors

Biosensors based on electrochemistry are extensively developed for bacterial detection because of their high sensitivity, rapidity and low cost. They can be classified as amperometric, voltametric, conductometric, potentiometric or impedimetric—the last two being the most used for foodborne pathogen detection. The use of peptides on electrochemical biosensors for the detection of foodborne pathogens was reviewed recently [22].

**Figure 4:**
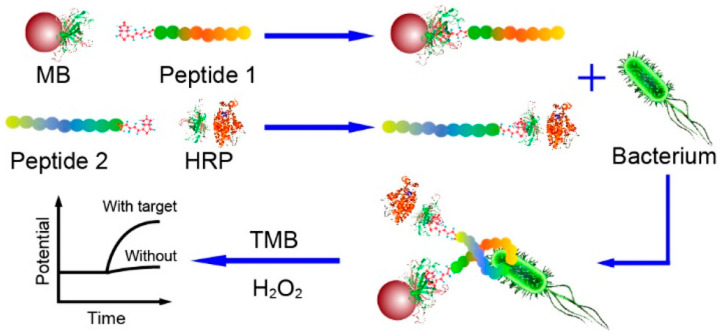
Schematic illustration of the potentiometric sandwich assay based on short antimicrobial peptide pairs for the detection of *L. monocytogenes* [118]. Copyright 2018, American Chemical Society.

Potentiometric sensing is based on the measurement of the potential differences between the working electrode and the reference electrode in the absence of an electrical charge flowing between them. Although it has many advantages, such as low cost, ease of use and rapidity, potentiometric biosensors require having control of the ionic strength of the sample. Otherwise, the different charges of species in the sample may interfere and lead to a potentiometric response, generating a false-positive result [117]. Lv et al. developed a potentiometric sandwich assay using short AMPs for the detection of *L. monocytogenes* in spiked seawater samples (Figure 4). For this, the original long AMP with a well-defined structure for *L. monocytogenes* was split into two fragments in order to serve as the peptide pairs for the sandwich assay. They succeeded in eliminating background interferences from the complex matrix and from other pathogenic bacteria with the addition of a magnetic separation step with Leucocin A-coated magnetic nanoparticles (MNPs) and the use of an online filtration system for the preconcentration of the target. The whole 60 min assay reached a limit of detection (LOD) of 10 CFU mL^−1^ without having a significant response to other bacteria, even those of the same Gram stain or of the same genus [118].

**Figure 5:**
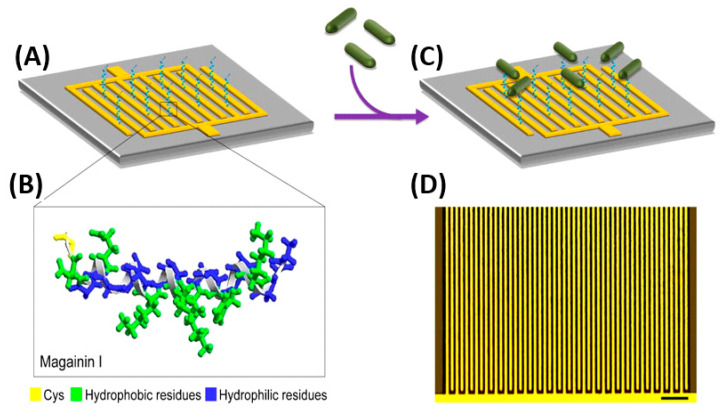
(**A**) Schematic impedimetric set up of Magainin I immobilized on an interdigitated microelectrode array. (**B**) Magainin I in helical form, modified with a terminal cysteine residue and with clearly defined hydrophobic and hydrophilic faces. (**C**) Detection of bacteria achieved via binding of target cells to the immobilized AMPs. (**D**) Optical image of the interdigitated microelectrode array (scale bar: 50 μm) [120]. Copyright 2010, National Academy of Sciences.

Electrochemical impedance spectroscopy (EIS) measures the impedance over a suitable frequency range through the application of a small sinusoidally varying potential. EIS biosensors offer simple instrumentation, ease of assembly and operation, adaptability to miniaturized devices and compatibility with multiplex detection [119]. These biosensors have achieved remarkably low LODs and linear detection ranges of up to six orders of magnitude for foodborne pathogens. Some of the first efforts for bacterial detection using AMPs were developed using EIS. Notably, Mannoor et al. immobilized the semi-selective AMP Magainin I onto an interdigitated gold electrode (GE) array via a C-terminal cysteine residue thanks to the formation of SAMs (Figure 5). Their microcapacitive biosensor demonstrated both Gram-selective detection, as well as interbacterial strain differentiation with detection limits of 1 × 10^3^ CFU mL^−1^, a clinically relevant detection range [120]. Since then, various breakthroughs in EIS biosensor performances have been achieved. Shi et al. put two phage display peptides specific to *E. coli* O157:H7 on a three-electrode system, capable of detecting 20 CFU mL^−1^ with only a 30 min incubation, which presents a remarkable improvement on LODs.

**Figure 6:**
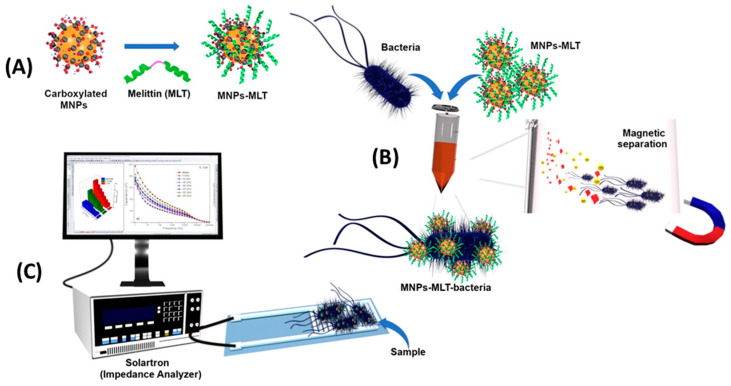
Schematic representation of the EIS biosensor based on magnetic nanoparticles functionalized with antimicrobial peptides. (**A**) Functionalization of MNPs with Melittin. (**B**) Capture of bacteria by MNPs-MLT, and magnetic separation of bacteria from the sample matrix. (**C**) EIS detection [121]. Copyright 2019, Elsevier.

Notably, Wilson et al. were able to detect *E. coli* with a LOD of 1 CFU mL^−1^ in potable water and 3.5 CFU mL^−1^ in apple juice without sample preparation and within only 25 min (Figure 6). First, they subjected the sample to a preconcentration step using magnetic nanoparticles coated with Melittin. Next, EIS measurements were performed using an interdigitated electrode array screen-printed onto the PET substrate as an inexpensive alternative to gold electrodes that require photolithography. Their system showed good repeatability and stability [121].

**Figure 7:**
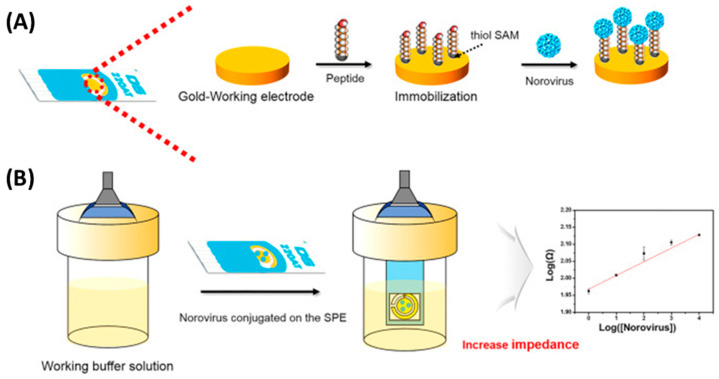
Schematic setup for norovirus detection using a peptide-based EIS biosensor. (**A**) The novel peptides were immobilized by the formation of SAMs on the Au working electrode. (**B**) Using a working buffer solution, the dropped norovirus on the Au working electrode was then measured, along with its strength affinity, by using an EIS analysis [40]. Copyright 2019, Elsevier.

In contrast, Baek et al. selected a much smaller target, the human norovirus. They immobilized eight norovirus-specific phage display peptides onto the screen-printed working electrode through the formation of SAMs (Figure 7). The obtained biosensors were able to detect copies 1.7 mL^−1^ from the oyster samples in 30 min without signal interference from another pathogenic species present, the rotavirus. This outstanding performance resulted in a biosensor much more sensitive than classical detection. Such a system provides a promising strategy for the identification and quantification of norovirus food contaminants with minimized sample preparations and volumes [40].

A common feature in EIS biosensors is the correlation of bacterial concentrations with impedance signals at low frequencies, which is an indication that impedance is related to charge transfer properties on the surface of the electrode. However, at higher frequencies bacterial concentrations show less influence on impedance, suggesting that, at that stage, the dielectric relaxation of small dipoles, including water molecules, becomes more dominant in impedance changes [120].

**Figure 8:**
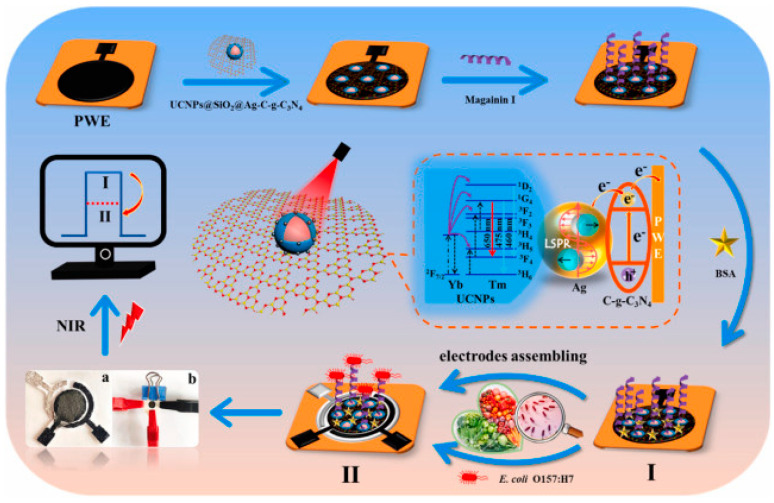
Schematic fabrication process of the PEC sensing platform for the detection of *E. coli* O157:H7. (I) Assembly of UCNPs@SiO_2_@Ag/C-g-C_3_N_4_, Magainin I and BSA onto PWE (II) PEC response of fabricated sensing platform under NIR light of 980 nm (a) photograph of modified PWE, Ag/AgCl reference electrode and carbon counter electrode assembled randomly on paper, (b) lab-on-paper PEC platform [122]. Copyright 2022, Elsevier.

Photoelectrochemical (PEC) techniques differ slightly from other electrochemical methods in that an applied light source generates electron excitation and charge transfer from a photoexcited material, which is semiconductive and converts visible light into a photocurrent. Yin et al. chose upconversion nanophores (UCNPs), a fluorophore able to transfer photon energy into luminescence to develop a PEC lab-on-paper platform triggered by near infrared (NIR) light for the detection of *E. coli* O157:H7 in food samples (Figure 8) [122]. NIR light is suited for biosensor use, as it possesses low phototoxicity, and better biocompatibility than ultraviolet (UV) light [123], which may result in serious interference and unstable signals [122]. Using Magainin I peptides as bioreceptors grafted onto paper working electrodes (PWE), the obtained biosensors demonstrated preferential binding to *E. coli* O157:H7, with the only mild interfering response obtained with *S. typhimurium*. They further improved the upconversion luminescence properties of their substrate by using silver nanoparticles (AgNPs) and exploiting their localized SPR (LSPR) effects, achieving the lowest limit of detection for Magainin I reported to date for this bacterium: 2 CFU mL^−1^, even when testing in complex food matrices.

### 3.2. Optical Peptide-Based Biosensors

Optical biosensors quantify analytes through the correlation of binding events with a measurable characteristic of light waves. They are often based on the measurement of absorbance, reflectance or fluorescence emissions that occur in the UV, visible or NIR light spectra [124]. Optical biosensors may either require labels, such as colorimetric or fluorescent approaches, or be label-free, such as biosensors based on SPR. The main advantages of these biosensors are reproducibility, sensitivity, the possibility of adaptation for multiplex detection and rapidity.

Labeled biosensors measure colorimetric or fluorescent changes that occur upon the interaction of a chromophore or fluorophore with the analyte. They consist of four elements: a light source, a wavelength selection device, a substrate in which changes will occur upon interaction with analytes and a detector sensitive to the wavelength of interest [125].

Fluorescence occurs when an electron is excited and a photon is emitted from an excited singlet state, and then, it relaxes to the ground state. This electron typically belongs to an aromatic molecule capable of producing fluorescence, called a fluorophore, which may be a dye, a product from an enzymatic reaction or a nanomaterial, such as nanoclusters (NCs) or quantum dots (QDs) [126]. Fluorescence is by far the most popular approach for optical detection due to its high sensitivity, as the emission of even a single photon may be sufficient to quantify it [127]. It is widely used in biosensing applications, as it is simple to set up, easily measured by fluorescence spectroscopy and it is normally the first proof-of-concept approach, such as in the case of ELISA immunoassays.

**Figure 9:**
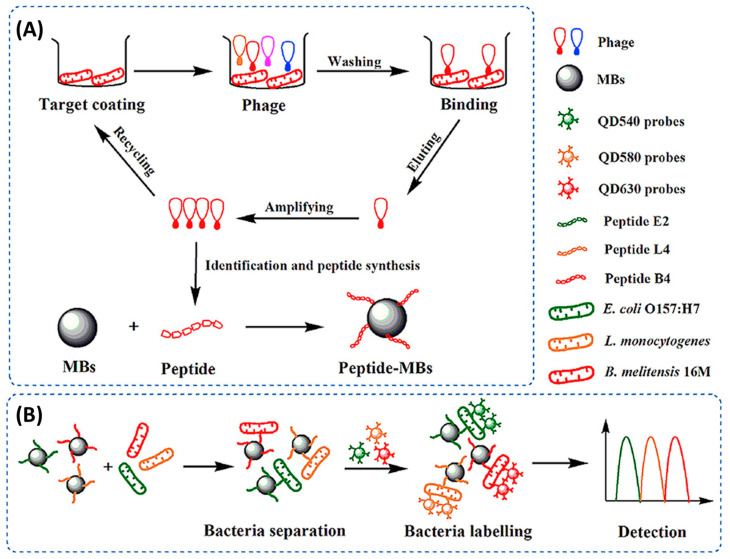
Schematic diagram of the simultaneous detection of *E. coli* O157:H7, *L. monocytogenes* and *B. melitensis* 16M based on phage-displayed peptides and the fluorescence spectroscopy set up [84]. Copyright 2020, Elsevier.

Some of the lowest limits of detection reported to date have been the result of the incorporation of phage display peptides onto optical biosensors, being two orders of magnitude lower than those reached when using AMPs. Li et al. achieved an optical biosensor for the simultaneous detection of three pathogens (*E. coli*, *L. monocytogenes* and *B. melitensis*) using phage display peptides and multicolor QDs. For this, peptides were immobilized onto magnetic beads (MBs) for the recognition and enrichment of targets from the complex sample matrix. Then, three QD probes with different emission wavelengths were functionalized with three polyclonal antibodies, respectively. By mixing the functionalized MBs and QDs, they obtained peptide MBs–pathogen–QD probes sandwich immune complexes, which allowed for the simultaneous fluorescence detection of three pathogens. Their highly sensitive and specific 100 min assay was able to differentiate and quantify the three foodborne pathogens (Figure 9) [84].

Colorimetric biosensors measure absorbance or reflectance events in the UV–Vis spectrum upon the interaction of chromophores with one or more analytes. These sensors often include nanomaterials, such as nanoparticles and nanosheets (NSs) as reporter structures [128]. Colorimetric platforms are commonly used for foodborne pathogen detection using peptides due to their versatility.

**Figure 10:**
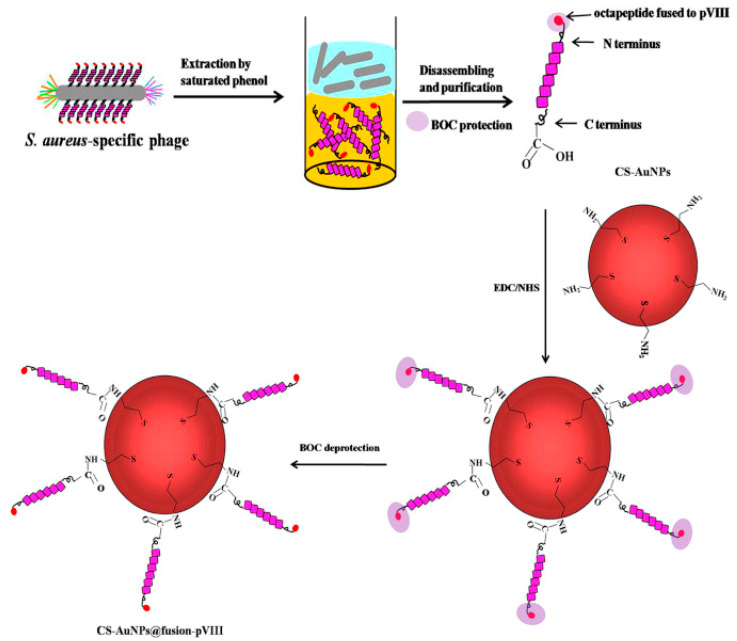
Schematic illustration of the isolation of the pVIII-coated protein fused with an *S. aureus*-specific octapeptide and the formation of CS-AuNPs@fusion-pVIII [86]. Copyright 2016, Elsevier.

Gold nanoparticle (AuNP)-based colorimetric assays have been widely used for biosensing, as they have unique surface plasmon resonance corresponding to their dispersion or aggregation state. Moreover, the concentration changes of targets can induce color changes visible to the naked eye. Liu et al. designed a colorimetric biosensor for the detection of *S. aureus* on various real water samples by immobilizing specific phage display peptides onto cysteamine-modified AuNPs (CS-AuNPs) (Figure 10). Such functionalized NPs aggregated quickly in the presence of the target *S. aureus* and were successfully used to detect the pathogen within 30 min with a LOD of 19 CFU mL^−1^ and excellent selectivity over other bacteria. This approach is particularly interesting due to its sensitivity, specificity and rapidity, with no need for any costly instrument [86].

**Figure 11:**
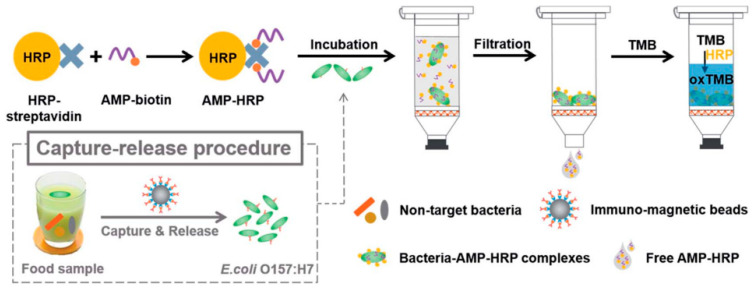
Schematic illustration of the AMP-based colorimetric bioassay for the detection of *E. coli* O157:H7 [130]. Copyright 2017, Royal Society of Chemistry.

Horseradish peroxidase (HRP) is an enzyme that is widely used in immunoassays such as ELISA due to its ability to catalyze the conversion of chromogenic substrates into colored products or produce light when acting on chemiluminescent substrates [129]. Qiao et al. bioconjugated AMP Magainin I with HRP through a biotin–streptavidin interaction for the rapid and extremely sensitive colorimetric detection of *E. coli* O157:H7 in apple juice and ground beef (Figure 11). The AMP–HRP conjugate, used as a signal reporter, bound to LPS on the surface of the Gram-negative bacteria, followed by a filtration step to reduce non-specific binding and steric effects. After which, the bacterial concentration could be easily visualized and quantified by UV–Vis absorption measurements. Their system could detect *E. coli* O157:H7 as low as 13 CFU mL^−1^ in a pure culture with a linear range of 10^2^–10^5^ CFU mL^−1^ in 45 min without pre-enrichment [130].

**Figure 12:**
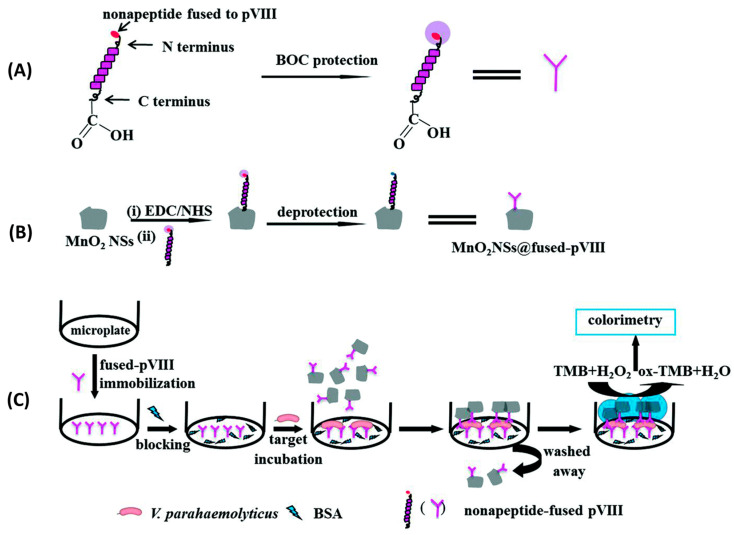
Schematic illustration of colorimetric detection of *V. parahaemolyticus*. (**A**) *V. parahaemolyticus*-specific pVIII fusion protected by tert-Butyl carbamate. (**B**) The C-terminal end of pVIII fusion was activated by EDC/NHS, bioconjugated with MnO_2_ NSs, and the N-terminal of pVIII fusion was deprotected. (**C**) MnO_2_NS@ pVIII fusion as sandwich immunoassay tags for *V. parahaemolyticus* detection [85]. Copyright 2018, Royal Society of Chemistry.

Although widely used in biochemistry, HRP has some limitations, such as high cost and low stability in some food matrices and over time. Consequently, there has been a surge in the search for stable, lower-cost inorganic nanomaterials with peroxidase-like activity. Such is the case of manganese dioxide nanosheets (MnO_2_NSs) used by Liu et al. to immobilize specific peptides for the detection of *Vibrio parahaemolyticus* in water and seafood samples (Figure 12). In this case, 9-mer phage display peptides were both fused to MnO_2_NSs to create a MnO_2_NSs@peptide complex and immobilized by physical adsorption onto a surface. In order to perform a sandwich immunoassay, bacteria were first incubated for two hours onto the peptide-grafted surface to ensure binding. Next, the MnO_2_NSs@peptide fusion was added for one hour to create the sandwich complex. Finally, chromogenic tert-Butyl carbamate (TMB) was added for 30 min, which resulted in color changes according to the bacterial concentration, determined by absorbance measurements at 652 nm. Their system showed a wide detection range (20–10^4^ CFU mL^−1^), a LOD of 15 CFU mL^−1^ and excellent selectivity. Finally, a practical performance was successfully demonstrated by spiking marine samples with recoveries from 98.0 to 102.5% [85].

As for label-free optical techniques, SPR-based sensing is commonly used for foodborne pathogen detection. SPR biosensors measure the changes in the refractive index in a dielectric medium due to the excitation of surface plasmons at the interface between said medium and a thin metal film, usually gold [131]. Its main advantages are its capability for real-time, label-free detection with high sensitivity.

**Figure 13:**
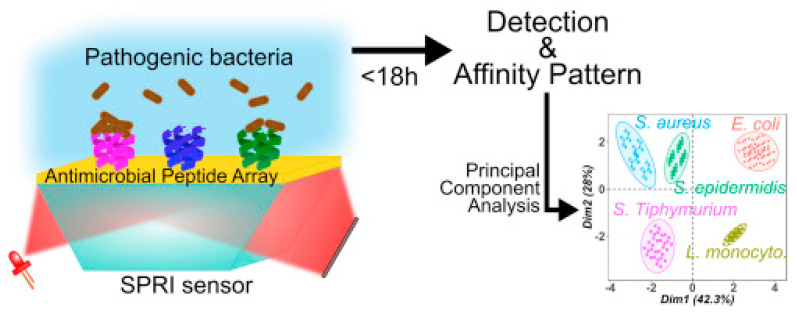
Schematic SPRI set up and principal component analysis discrimination between *S. aureus*, *S. typhimurium*, *S. epidermis*, *E. coli* and *L. monocytogenes* [111]. Copyright 2019, Elsevier.

Surface plasmon resonance imaging (SPRI) is a multiplex SPR approach based on an imaging mode. It allows for simultaneous monitoring of the interactions between the analyte and hundreds of sensors on the same chip with a temporal response and kinetic information, which may provide additional discriminatory parameters [132]. Pardoux et al. developed a prism coupler-based SPR biosensor using a five AMP microarray for the detection of pathogenic bacteria. The detection of five different pathogens by SPRI can be achieved in an 18 h single step, as it is a label-free technique in which no pre-enrichment is required. In this case, the wide-spectrum recognition of AMPs was particularly relevant, as the differing levels of affinity characteristic of these peptides created a cross-reactive sensor matrix that, coupled with multivariate analyses, was able to accurately discriminate between bacteria (Figure 13). Furthermore, they achieved some of the lowest LODs for *E. coli* O157:H7, *S. epidermis* and *S. typhimurium*, detecting 51, 16 and 6 CFU mL^−1^, respectively [111].

**Figure 14:**
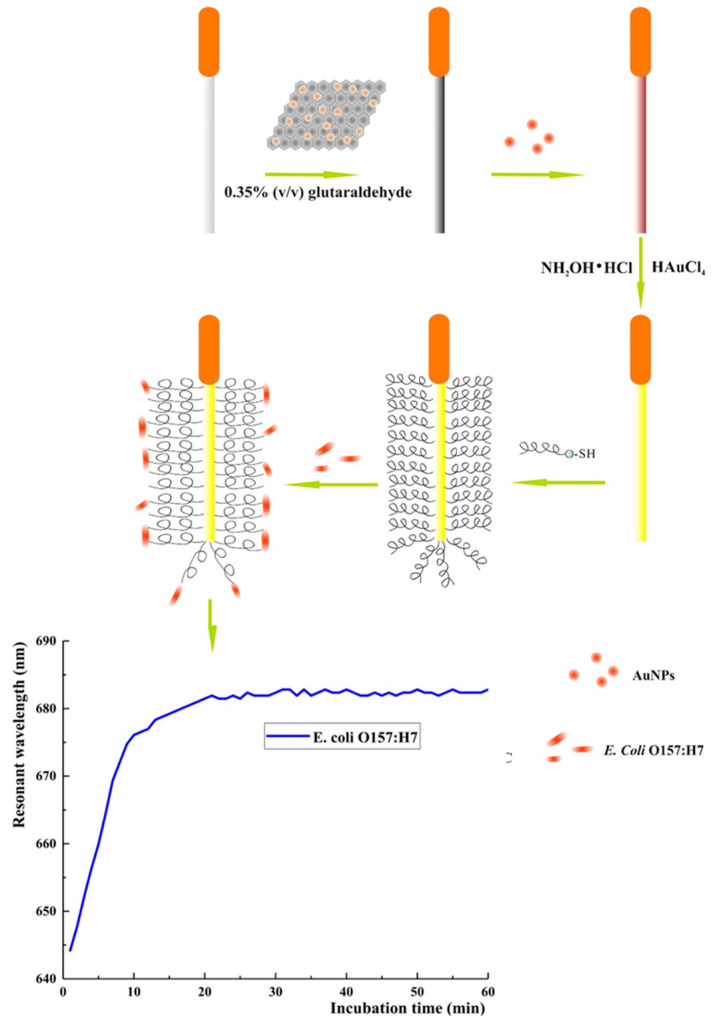
Schematic illustration of optical fiber SPR detection of *E. coli* O157:H7 **[114]**. Copyright 2018, Elsevier.

Zhou et al. developed a wave guide coupler-based SPR biosensor using optical fibers for the detection of pathogenic Gram-negative *E. coli* O157:H7 in water and juice using Magainin I as a bioreceptor and AgNP-reduced graphene oxide (AgNP-rGO) nanocomposites for signal amplification (Figure 14). The biosensor had a LOD of 5 × 10^2^ CFU mL^−1^ and showed little to no interference of nonpathogenic or Gram-positive bacteria present in the sample and remarkable reproducibility, obtaining a 4.2% relative standard deviation (RSD) in five biosensors constructed in parallel [114].

**Figure 15:**
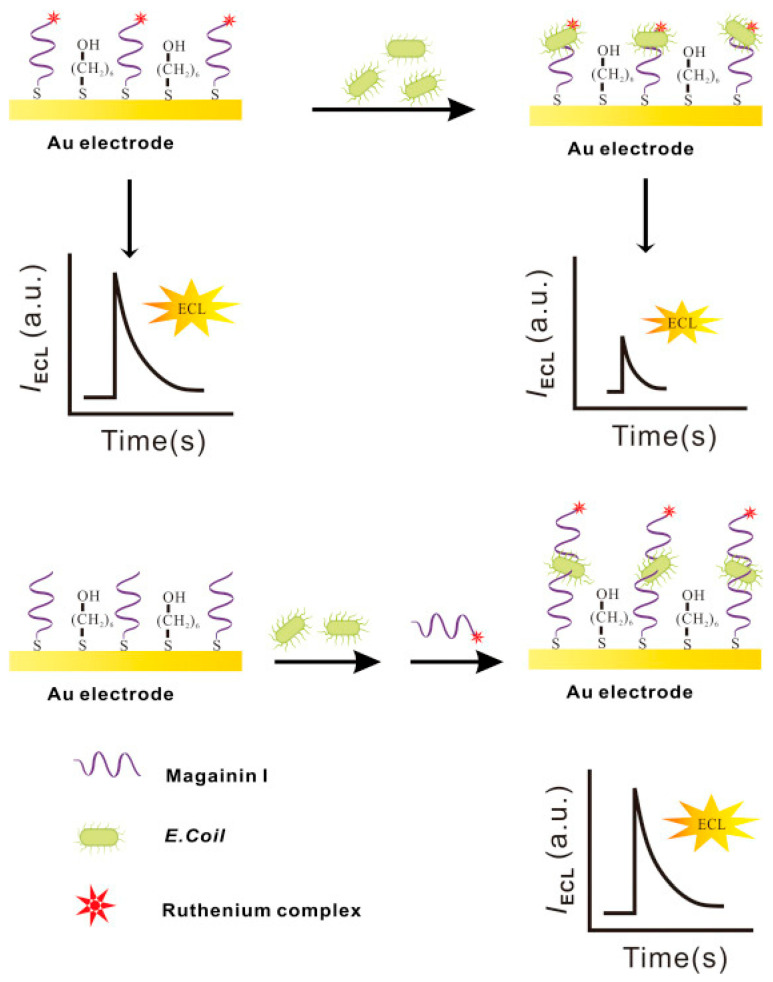
Schematic electrochemiluminescence biosensor fabrication and ECL determination of *E. coli* O157:H7 [133]. Copyright 2015, Elsevier.

Electrochemiluminescence (ECL), contrary to photoelectrochemistry, consists of monitoring the production of photons, namely the light intensity produced during the electrochemical reaction in a solution. This analytical method provides outstanding benefits: excellent sensitivity due to the absence of background noise, versatility, spatial and temporal resolution and electrochemical control of the reactivity. Li et al. incorporated Magainin I into an ECL platform in a sandwich assay for the highly specific detection of *E. coli* O157:H7 in water. They immobilized Magainin I onto the gold working electrode surface as a bioreceptor. Additionally, they labeled the peptide with a ruthenium complex (Ru1) ECL label, which increases the ECL intensity proportionally to the increasing bacterial concentrations in the sample. Their biosensor, which did not need any pre-enrichment or separation steps, achieved a LOD of 1.2 × 10^2^ CFU mL^−1^ and allowed Magainin I to keep its characteristic selectivity towards Gram-negative bacteria (Figure 15). However, it was not able to discriminate between pathogenic *E. coli* O157:H7 and *S. typhimurium* [133].

### 3.3. Nanomechanical Peptide-Based Biosensors

**Figure 16:**
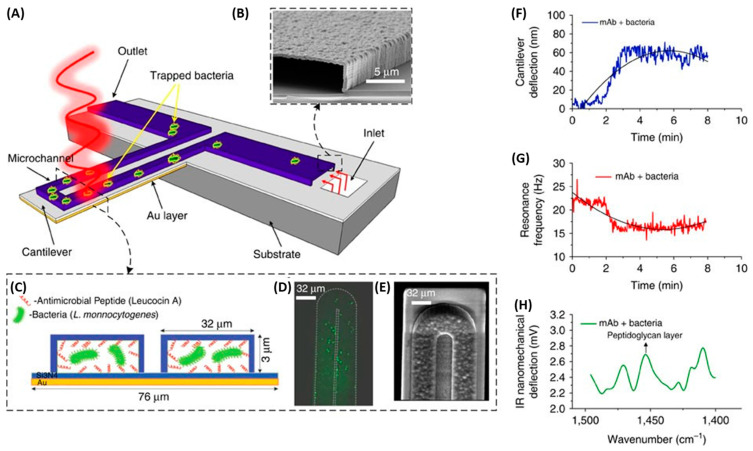
The schematic illustration of the nanomechanical peptide-based biosensors and its multi-mode of operation (**A**) BMC filled with bacteria supported on a silicon substrate. The BMC was coated with a bacteria-targeted receptor and irradiated with a specific wavelength of tunable infrared light. (**B**) Scanning electron microscopy (SEM) image of the cross-section of an inlet, through which an aqueous solution of bacteria is loaded. (**C**) Cross-section of the 32 μm wide microchannel of the cantilever functionalized with either a mAb or Leucocin A, which acted specifically against *L. monocytogenes*. (**D**) Fluorescent image from the top side of the BMC, filled with bacteria. (**E**) SEM image of the tip of the BMC. (**F**) When the bacteria inside the BMC absorbs infrared light, local heat is generated that results in the nanomechanical deflection of the BMC. (**G**) The resonance frequency is sensitive to the increased mass caused by the adsorption of bacteria inside the BMC. (**H**) When the BMC is illuminated with a certain range of infrared light, a plot of the nanomechanical deflection of the BMC shows the wavelength where the bacteria absorb infrared light [135]. Copyright 2016, Springer Nature.

Mechanical biosensors are based on the measurement of forces, displacements and mass changes [134]. Most mechanical biosensors have a small cantilever sensitive to the molecule of interest. The microcantilever translates binding events into mechanical signals by monitoring deflection changes. Etayash et al. developed a microfluidic channel on a biomaterial cantilever (BMC) to detect *L. monocytogenes* functionalized with anti-*L*. *monocytogenes* monoclonal antibody and AMP Leucocin A in only a 50 picoliter volume (Figure 16). Bacterial adsorption induced changes in the resonance frequency and cantilever deflection. When exciting the trapped bacteria with infrared radiation, the cantilever deflected in proportion to the infrared absorption of the bacteria, providing a nanomechanical infrared spectrum for selective bacterium identification. The Leucocin A-coated BMC exhibited preferential binding to *L. monocytogenes* two to three orders of magnitude higher than to *E. coli*. Furthermore, they achieved a limit of detection of 100 cells in 100 µL water samples. Through the incorporation of infrared absorption spectroscopy, they were able to accurately differentiate between injured and intact cells [135].

Table 4 summarizes peptide-based biosensors using various transduction systems together with their performances. Clearly, the excellent stability and low production cost make peptides very promising bioreceptors compared to antibodies. Most importantly, the performances of the obtained peptide-based biosensors are remarkable.

Although various breakthroughs have been achieved, and in some cases, the biosensor performance is already comparable to that of the classical techniques or immunoassays, key challenges remain in foodborne pathogen detection biosensors. The main ones often concern the complexity of the food matrix itself due to its diverse composition, as well as the electrical charge of said components. In this media, the accurate detection of bacterial species might be especially challenging for peptide interactions that are dominated by electrostatic interactions. As an example, Etayash et al. succeeded in the discrimination of multiple species of pathogenic Gram-positive bacteria in buffer solutions. However, the results were not the same when working with pure milk samples, possibly due to the high protein composition of the sample [136]. Another major challenge is cross-contamination from other microorganisms. When a biosensing platform is developed for a specific application, it is important to screen against all typically cross-reactive species in that particular ambit in order to validate its applicability, which several reported biosensors have failed to do [143,145]. To address the inability of peptides to account for cross-contaminating dead bacteria, Fan et al. coupled their detection technique with a luciferase bioluminescence system to quantify ATP, a molecule only found in live organisms [144].

Furthermore, there are varieties of proteases in different foods, especially unprocessed foods, which can degrade peptides into smaller molecules or single amino acids and inactivate them. These proteases, such as trypsin, thermolysine or carboxypeptidases, are one of the major limitations preventing the real-life application of peptide-based biosensors. However, the stability of peptides may be increased through chemical modifications that prevent enzymatic degradation, including click chemistry application to stabilize peptide dimerization or multimerization [148], replacement of an L-enantiomer by its D-enantiomer [149] and conjugation of specific groups such as fatty acids or side-chain analogs to peptide side chains or N- or C-terminals [150,151]. These fine-tunings make it difficult for proteases to recognize the cleavage sites, providing to the peptide a prominent proteolytic resistance. However, chemical modifications may decrease or inactivate the peptide recognition efficiency and stabilized peptides’ binding analytical properties must be tested before their implementation.

## 4. Emerging Peptide-Based Electronic Noses for Foodborne Pathogen Detection

Limitations in classical sensing technologies have resulted in a surge in the exploration of innovative, nonconventional methodologies. In parallel with the development of biosensors, other sensor-based technology is emerging. A notable example is electronic nose, which takes a completely different approach for detecting the presence of pathogenic bacteria by analyzing their emitted volatile organic compounds (VOCs).

Indeed, bacteria produce and emit VOCs that play a vital role in inter and intraorganismal communication. They may serve as signal molecules between species, chemical ‘manipulators’ to alter metabolic pathways, contribute to nutrient scavenging or participate in developmental processes [152]. The bacterial headspace, referring to the gaseous mixture above a bacterial culture, has been the basis for microorganism identification, as VOCs can be species-specific [153]. This type of detection is beginning to be explored due to its potential applications in the diagnosis of infectious diseases in humans, and great efforts have been made to characterize the VOC composition of patients’ exhaled breath, saliva, urine and feces at various states of health [154,155,156]. In the food industry, efforts to detect specific VOCs indicative of freshness, adulteration and foodborne pathogen contamination at trace levels are ever growing, whether they are in food samples themselves, during the processing stages or in their packaging. Therefore, electronic noses could be relevant alternatives for foodborne pathogen detection [157,158,159]. Since eNs require no sample preparation, they can be used to analyze and screen foodstuffs in all phases of production.

Electronic noses are a broad class of instruments constituted by an array of chemical sensors with a partial specificity to VOCs coupled to a pattern–recognition system that detects and identifies odors [160]. Their response to VOCs is a distinct and unique fingerprint-like recognition pattern usually stored in a database, which acts as a reference library to which future samples will be compared. These systems were inspired by the biological sense of smell, in which the sensation of smell is produced upon the binding of VOCs emitted by an object to odorant-binding proteins (OBPs), which relay the aromatic molecules onto olfactory receptors (ORs) located in the nose [161]. Afterwards, olfactory neurons convey the received signal to the cortex of the brain, which oversees signal processing and interpreting for the identification of specific odors.

**Figure 17:**
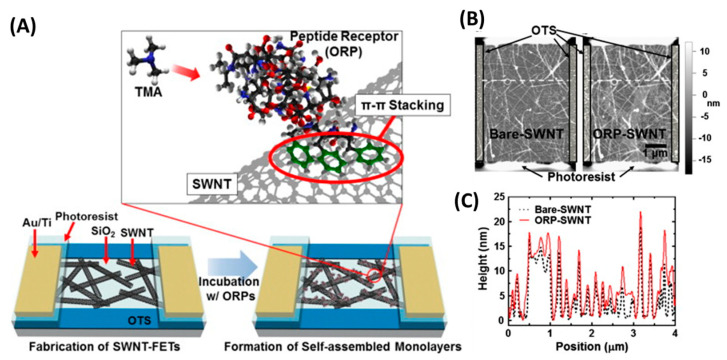
The electronic nose for the detection of TMA. (**A**) ORPs were self-assembled on the surface of SWNTs during the treatment of ORP-suspended deionized water solutions. The ORPs were immobilized by π–π stacking of aromatic rings at their C-terminus and attracted TMA molecules very near to the SWNTs. (**B**) Atomic force microscopy images of bare and ORP-immobilized SWNT channels on the fabricated sensor devices. (**C**) Height profiles of specific cross sections (white dashed lines in (**B**)). The height of SWNTs increased by 2–3 nm after immobilization of the ORPs. [162]. Copyright 2013, Elsevier.

Several groups have developed peptide-based eNs for foodborne pathogen detection. For example, the group of T.H. Park used a peptide derived from a natural olfactory receptor that can specifically recognize trimethylamine (TMA) to monitor seafood spoilage [162]. TMA is an indicator VOC whose concentration in seafood increases after death due to the decomposition of trimethyl-N-oxide. In this case, single-wall carbon nanotube field effect transistors (SWCNT-FETs) functionalized with olfactory receptor-derived peptides (ORPs) were used to selectively detect TMA at a concentration of 10 fM in real time without sample pretreatment and with excellent selectivity (Figure 17). Furthermore, the eN was able to discriminate between spoiled seafood and other food samples.

**Figure 18:**
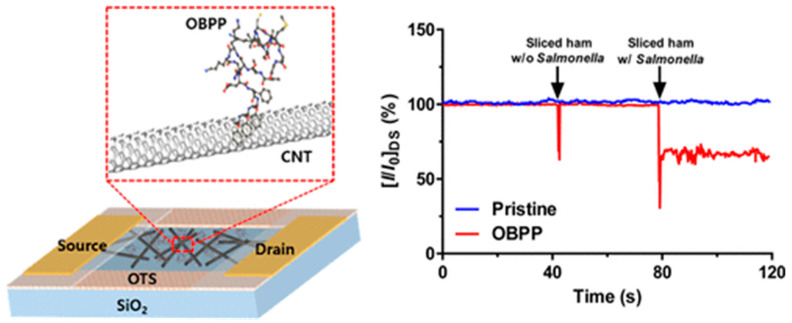
Schematic setup of CNT-FET for the detection of *Salmonella* contamination in ham and real-time detection of *Salmonella* contamination in sliced ham [165]. Copyright 2016, American Chemical Society.

Sankaran et al. synthetized a polypeptide derived from a *Drosophila* OBP named LUSH [163]. The chosen 14-mer peptide included the protein’s sensing domain known to bind preferentially to alcohols, such as 3-methyl-1-butanol and 1-hexanol, characteristic odorants of *Salmonella* contamination. Four peptide receptors were grafted onto a QCM through the formation of SAMs. When testing packaged beef, they were able to detect 1 ppm of 3-methyl-1-butanol and 1-hexanol, a relevant LOD for industrial applications, with good repeatability and reproducibility [164]. Similarly, Son et al. employed a 20-mer peptide derived from LUSH protein’s binding domain to detect *Salmonella* contamination in ham using a carbon nanotube field effect transistor (CNT-FET). They immobilized the peptide onto CNTs by π–π stacking through the addition of three phenylalanine amino acids at the C-terminus. Their system was able to detect 3-methyl-1-butanol at a concentration of 1 fM in real time (Figure 18) [165].

In a recent example, Shumeiko et al. succeeded in distinguishing between the odor of sterile growth medium, *E. coli* and *Klebsiella pneumoniae* by incorporating peptide-functionalized SWCNTs to a low-cost NIR photoluminescence optical nose for the detection of these species’ indicator VOCs. When dispersed in aqueous solutions, SWCNTs emit photoluminescence upon excitation with an appropriate wavelength [166]. In this case, they used five peptides based on their ability to disperse SWCNTs in water and the resulting photoluminescence intensity. Upon the 60 s exposure of the sensor to *E. coli* and *K. pneumonia*, none of the five receptors were able to differentiate between sterile and spiked mediums. However, the accurate discrimination of the samples was achieved upon analyzing the recovery kinetics of the sensors, highlighting the crucial role of data processing in electronic noses [167].

It is clear that the recent interest in developing eN platforms for bacterial detection has resulted in extremely sensitive instruments capable of real-time monitoring. However, the development of eNs with greater selectivity towards VOC targets may result in great breakthroughs. To this end, some attempts have been made at the adaptation of phage display panning for screening specific peptides for gas sensing, especially for the detection of explosives [168], but to the best of our knowledge, none has been used for food quality assessment or foodborne pathogen detection.

## 5. Conclusions and Outlook

Biosensor technologies are very promising for the development of alternatives for pathogen detection with high sensitivity, low cost, rapid response and potential portable devices for an on-site analysis. The use of peptides as bioreceptors in biosensors is a growing field due to their versatility, increased stability in harsh conditions compared to other biomolecules, their compatibility with biosensor construction by maintaining their activity even after being dried and the possibility of finding or designing peptide sequences with affinities similar to those of antibodies. Furthermore, compared to the classical detection methods, one of the main advantages of peptide-based biosensors and electronic noses is that they bypass complex sample preparation, the most time-consuming and expensive step of foodborne pathogen detection, as their targets are either the bacterial surface epitopes or the emitted VOCs present in the headspace instead of the intracellular biomarkers.

Multiple selection strategies have resulted in the creation of sensitive and selective peptide bioreceptors, each with their own advantages. AMPs have been part of living organism’s defense systems for millennia and mostly rely on membrane disruption and blocking metabolic functions of competing microorganisms. Most AMPs have a selectivity towards a certain type of microorganism, be it Gram-positive or -negative bacteria, yeasts or fungi. Additionally, their longevity as defenders of living organisms, and their mechanism of action, decrease the possibility for targeted microorganisms to develop resistance against them. Alternatively, phage display is one of the most notable recent developments in the field, achieving the screening of millions of peptide candidates for the selection of a few highly selective probes. The incorporation of phage display peptides has resulted in extremely sensitive biosensors able to discern in between serovars or strains of the same species, a feat AMPs are unable to perform for the most part. However, this strategy is limited by the fact that the presentation of the target to the peptides displayed by phages is determining, which can be especially challenging when dealing with bacterial targets, due to the complexity of the bacterial membrane. In the most recent years, the availability of bioinformatics tools has resulted in the development of much faster screening processes and are promising alternatives when screening using biological methods, such as phage display, is not feasible. These strategies are especially advantageous because they allow the user to explore millions of candidates in silico without having to synthetize them, making them a more cost-effective option. Furthermore, these approaches can be combined for better peptide selection, for example, by using known AMPs as starting templates to construct chimeric peptides with enhanced selectivity or iterating specific motifs known to confer given physicochemical or structural characteristics or further refine the specificity of peptides selected by phage display.

Various peptide-based biosensors and electronic noses have been successfully developed for foodborne pathogen detection with good performances. For the future, in the age of miniaturization, there is a clear tendency in the biosensor field towards portable technologies, such as the use and integration of microfluidic devices. These devices pass extremely low sample volumes, down to pico-liter levels, through microchannels, usually designed computationally and fabricated with polymers using soft lithography [116,120,135,146,147]. Although it is well suited for on-field applications, the extremely low volume of analysis may present a limitation. To ensure efficient detection, microfluidic devices may be used in a preconcentration step in order to obtain a smaller volume with a higher concentration, which may then be detectable with conventional methods. Regarding transduction techniques, there have been several breakthroughs. For example, in electrochemical transduction, the use of screen-printed electrodes and the development of photoelectrochemical biosensors have greatly improved the efficiency of detection. As for optical transducers, SPRI has proven to be a reliable approach, as it provides excellent sensitivity with the possibility to make multiplex detections simultaneously and provide kinetic parameters, which may result in improved discrimination. Finally, hybrid methodologies, such as electrochemiluminescence, show great promise due to their simple optical set up and versatility and exceptional sensitivity.

Furthermore, the addition of nanomaterials to the sensing components for signal enhancement is a trend that has resulted in enormous improvements in foodborne pathogen biosensors. Nanomaterials have been incorporated into various types of transducers due to their capability of amplifying detection signals, which is a crucial factor for reaching a higher sensitivity. Recent works have demonstrated that the full potential of nanomaterials, such as nanoparticles, nanosheets, nanoclusters and quantum dots, is just beginning to be explored in depth, especially concerning their role in enhancing the performance of existing detection strategies [86,114,118,122,138,144,145]. Finally, the commercial success of any one of these developed biosensors depends on their ability to reliably improve one of the major limitations of the classical techniques (i.e., detection time, portability, sensitivity or a combination of the aforementioned) while still being economically viable to implement at the industrial level.

## Figures and Tables

**Table 1:** Characteristics of the top foodborne pathogens worldwide and EU regulatory limits in foodstuffs [1,5].

Foodborne Pathogen	Classification and Characteristics	Top Food Vehicles	Current Detection Methodologies	EU Regulatory Limit in Foodstuffs
*Escherichia coli* O157:H7	- Gram-negative - Rod-shaped - Facultative anaerobic - Non-spore forming - Toxin-producing	- Tap water - Well water - Cheese - Dairy products - Red meat and products	- Enrichment in selective media - Culture isolation on agar plates - Detection and identification of toxins by colony blot	0 CFU in 25 g of food
*Listeria monocytogenes*	- Gram-positive - Rod-shaped - Facultative anaerobic - Non-spore forming	- Fish and products - Red meat and products - Cheese	- Enrichment in selective media - Culture isolation on agar plates - PCR - RT-PCR	100 CFU/g
*Campylobacter jejuni*	- Gram-negative - Curved or spiral - Microaerophilic - Non-spore forming	- Broiler meat and products - Milk	- Enrichment in selective media - Culture isolation on agar plates - PCR - MALDI-TOF	1000 CFU/g on broiler carcasses
*Salmonella* spp. (*S*. *enterica* Enteritidis, *S. enterica* Typhimurium)	- Gram-negative - Bacilli - Relatively anaerobic - Non-spore forming	- Eggs and products - Pig meat and products - Bakery products	- Enrichment in selective media - Culture isolation on agar plates - Serotyping - RT-PCR	0 CFU in 25 g of product in the market
Norovirus	- Icosahedral - Non-enveloped - RNA viral particle	- Fruits and vegetables - Seafood	- RT-PCR	No regulatory limit

**Table 2:** Characteristics of natural antimicrobial peptides used in biosensors for foodborne pathogen detection [42].

Peptide	Sequence	Net Charge	3D Structure	Organism of Origin	Selectivity	Ref.
Magainin I	GIGKFLHSAGKGKAFVGEIMK	3	Unknown/too flexible	*Xenopus laevis*	Anti-Gram-positive Anti-Gram-negative Antiviral	[45]
Clavanin A	VFQFLGKIIHHVGNFVHGFSHVF	5	Helical	*Styela clava*	Anti-Gram-positive Anti-Gram-negative Antifungal	[46]
Nisin A	ITSISLCTPGCKTGALMGCNMKTATCNCSIHVSK	3	Beta sheet	*Streptococcus lactis*	Anti-Gram-positive Spermicidal Anticancer	[56]
Cecropin A	KWKLFKKIEKVGQNIRDGIIKAGPAVAVVGQATQIAK	6	Unknown	*Heliothis virescens*	Anti-Gram-positive Anti-Gram-negative Antifungal	[57]
Pediocin	KYYGNGVTCGKHSCSVDWGKATTCIINNGAMAWATGGHQGNHKC	3	Helical and Beta sheet	*Pediococcus acidilactici*	Anti-Gram-positive Spermicidal	[58]
Leucocin A	KYYGNGVHCTKSGCSVNWGEAFSAGVHRLANGGNGFW	2	Helical and Beta sheet	*Leuconostoc gelidum * UAL-187	Anti-Gram-positive Anti-Gram-negative	[59]
Protonectin	ILGTILGLLKGL	2	Helical	*Protonectarina sylveirae*	Anti-Gram-positive Anti-Gram-negative	[60]
Plantaricin-423	KYYGNGVTCGKHSCSVNC	6	Helical	*Lactobacillus plantarum*	Anti-Gram-positive Anti-Gram-negative Anticancer	[61]
Colicin V	ASGRDIAMAIGTLSGQFVAGGIGAAAGGVAGGAIYDYASTHKPNPAMSPSGLGGTIKQKPEGIPSEAWNYAAGRLC	0	Unknown	*Escherichia coli*, *Homo sapiens* microbiota	Anti-Gram-negative	[62]
Warnericin RK	MQFITDLIKKAVDFFKGLFGNK	2	Helical	*Staphylococcus warneri RK*	Anti-Gram-negative	[63]
Curvacin A	ARSYGHGVYCNNKKCWVNRGEATQSIIGGMISGWASGLAGM	3	Helical and beta sheet	*Lactobacillus sake* Lb706	Anti-Gram-positive	[64]
Melittin	GIGAVLKVLTTGLPALISWIKRKRQQ	6	Helical	*Apis mellifera*	Anti-Gram-positive Anti-Gram-negative Antiviral Antifungal Antiparasitic	[65]

**Table 3:** Peptides selected by phage display for foodborne pathogen detection.

Amino Acid Sequence	Target	Biopanning Strategy	Phage Display Library	Bacteria Exposed	Gram Stain	Selectivity Assessment Technique	Selectivity	Ref.
SEAYKHRQMHMSGGGSC NRPDSAQFWLHH VPWVTTYEPWGM GPADNTSKHVIR	*Salmonella* spp. cocktail (8 serovars)	Surface immobilized	Ph.D.-12	*Salmonella* spp. *L.monocytogenes* *E. coli* *C. jejuni*	- + - -	ELISA	5× greater for *Salmonella* spp.	[76]
QRKLAAKLT	*P. aeruginosa*	In solution	pVIII-9aa pVIII-12aa	*P. aeruginosa*, *S. typhimurium* *S. flexneri* *L. monocytogenes* *B. subtilis*	- - - + +	ELISA High-power optical phase contrast microscopy	10× greater for *P. aeruginosa*	[77]
RVRSAPSSS	*Staphylococcus aureus*	In solution	pVIII-9aa	*S. epidermis* *L. monocytogenes* *B. subtilis* *E. coli* *P. aeruginosa* *S. flexneri*	+ + + - - -	ELISA	4× greater for *S. aureus*	[78]
FLIDSPLASIGPTSM FMIDSPLASIGPTSM FLSDPPAPPTSPGVV	*C. jejuni*	In solution	f88-4/15-mer	*C. jejuni*	-	-	-	[79]
VTPPTQHQ	*S. typhimurium*	In solution Surface immobilized	f8/9	*S. typhimurium* *P. aeruginosa* *E. aerogenes* *C. freundii* *K. pneumonieae* *S. flexneri* *E. coli* *Y. enterocolitica* *S. marcescens* *P. mirabilis*	- - - - - - - - - -	ELISA (phage-capture, *Salmonella* capture) Precipitation assay Fluorescent microscopy Electron microscopy	10–1000× greater for *S. typhimurium*	[80]
QHIMHLPHINTL	Norovirus	-	Ph.D.-12	*-*	-	ELISA	-	[81]
LPSWYLAYQKII	Norovirus	-	Ph.D.-12	*-*	-	ELISA	-	[7]
AWLPWAK NLQEFLF	*S.* Enteritidis LPS	Immobilized on epoxy beads.	Ph.D.-7	*S. enteritidis* *S. typhimurium* *S. typhosa* *E. coli* K-235 *E. coli* O111:B4 *P. aeruginosa*	- - - - - -	ELISA Confocal microscopy	-	[82]
VPHNPGLISLQG	*S. aureus*	Surface immobilized	P.h.D.-12	*S. aureus* *S. epidermis* *E. coli* *P. aeruginosa* *K. pneumonia* *B. cereus*	+ + - - - +	ELISA Dot-blot assay Fluorometry	7× greater for *S. aureus*	[83]
SLLTPVP MWPHPLY DNDLSLS	*E. coli* O157:H7 *L. monocytogenes* *Brucella melitensis* 16 M	Surface immobilized	Ph.D.-7	*E. coli* O157:H7 *L. monocytogenes* *B. melitensis* 16 M *S. typhimurium* *S. aureus* *S. faecalis* *C. braakii* *S. boydii* *E. cloacae* *P. mirabilis* *E. sakazakii* *K. pneumonia*	- + - - + + - - - - - -	ELISA	2× greater for *E. coli* 7× greater for *L. monocytogenes* 2× greater for *B. melitensis*	[84]
VQTVQIGSD	*Vibrio parahaemolyticus*	Surface immobilized	f8/8	*V. parahaemolyticus* *E. coli* DH5α *S. aureus* *P. vulgaris* *E. tarda* *V. anguillarum*	- - + - - -	Phage recovery count	30× greater for *V. parahaemolyticus*	[85]
GQTTLTTS DPKTLNST EPRTPSPT	*Staphylococcus aureus*	Surface immobilized	f8/8	*S. aureus* *B. cereus* *E. tarda* *E. coli* *P. vulgaris* *V. parahemolyticus* *V. anguillarum*	- + - - - - -	Phage capture assay	-	[86]
VVSPDMNLLLTN GLHTSATNLYLH	*Escherichia coli* O157:H7	In solution	Ph.D.-12	*E. coli* O157:H7	-	ELISA	3× greater for *E. coli*	[87]
GRIADLPPLKPN	*L. monocytogenes*	In solution Surface immobilized	Ph.D.-12	*L. monocytogenes* *L. innocua* *Salmonella* spp. *E. coli* *C. jejuni*	+ + - - -	ELISA (Phage-binding) Magnetic separation	43× greater for *L. monocytogenes*	[88]

**Table 4:** Peptide-based biosensors for foodborne pathogen detection.

Transduction System	Exposed Pathogen	Peptide (Type)	Immobilization Technique	Food/Water Sample	LOD (CFU/mL)	Linear Range (CFU/mL)	Analysis Time (min)	Ref.
Electrochemical impedance spectroscopy	*E. coli* O157:H7	GLHTSATNLYLHGGGC (phage display)	Covalent binding	PBS	20	2 × 10^2^–2 × 10^6^	30	[87]
Electrochemical impedance spectroscopy	*E. coli* O157:H7 *E. coli* K12 *S. epidermis* *B. subtilis*	Magainin I (AMP)	Covalent binding	PBS	10^3^	10^3^–10^7^	90	[115]
Electrochemical impedance spectroscopy	Norovirus	QHKMHKPHKNTKGGGGSC (phage display)	Covalent binding	Oyster	1.7 copies/mL	1–10^5^ copies/mL	30	[40]
Electrochemical impedance spectroscopy	*L. monocytogenes*	Leucocin A (AMP)	Covalent binding	Milk (10–100% in PBS)	10^3^	10^3^–10^6^	Real-time	[136]
Electrochemical impedance spectroscopy	*S. typhimurium*	Melittin (AMP)	Covalent binding	Potable water Apple juice	1 3.5	1–10^6^	25	[121]
Electrochemical impedance spectroscopy	*E. coli* *L. monocytogenes* *S. typhimurium*	Magainin I (AMP)	Covalent binding	PBS	10^3^	10^2^–10^7^	Real-time	[120]
Electrochemical impedance spectroscopy	*E. coli* O157:H7	Colicin V (AMP)	Covalent binding	Water	10^2^	10^2^–10^6^	15	[137]
Electrochemical impedance spectroscopy	*K. pneumoniae* *P. aeruginosa* *Enterococcus faecalis* *Candida tropicalis*	Synoeca-MP (AMP)	Covalent binding	PBS	10 10 10 10	10–10^5^	5	[138]
Electrochemical impedance spectroscopy	*K. pneumoniae* *Enterococcus faecalis* *E. coli* *B. subtilis*	Clavanin A (AMP)	Covalent binding	PBS	10^2^	10^2^–10^6^	-	[139]
Electrochemical impedance spectroscopy	*Salmonella* spp. *E. coli* O157:H7 *Listeria innocua*	Nisin (AMP)	Covalent binding	Milk	1.5 × 10^1^	1.5 × 10^1^–1.5 × 10^4^	30	[140]
Electrochemical impedance spectroscopy	Norovirus	QHKMHKPHKNTKGGGGSC (phage display)	Covalent binding	Oyster	1.7 copies/mL	0–10^5^ copies/mL	90	[40]
Electrochemical impedance spectroscopy	*P. aeruginosa* *S. mutans*	C16G2cys (Synthetic) G10KHC (Synthetic)	Covalent binding	LCB PBS	10^5^	10^4^–10^7^	25	[141]
Potentiometry	*L. monocytogenes* *Listeria iuanuii* *S. typhimurium E. coli* O157:H7	Leucocin A (AMP) Leucocin A14 (AMP)	Streptavidin- Biotincoulping to MBs	Seawater	10	10^2^–10^6^	60	[118]
Photoelectrochemistry (NIR)	*E. coli* O157:H7	Magainin I (AMP)	Cross-linking	Pork Cabbage Milk	2	5–5 × 10^6^	50	[122]
Colorimetry	*E. coli* O157:H7 *V. parahaemolyticus* *S. typhimurium L. monocytogenes* *E. coli* DH5α *S. aureus*	Magainin I (AMP)	Streptavidin-Biotin coupling to HRP	Apple juice Ground beef	13	10^5^–10^7^	45	[130]
Colorimetry	*S. aureus*	GQTTLTTS (phage display)	Covalent binding	Drinking water Tap water River water Sewage	19	20–2000	30	[86]
Colorimetry	*V. parahaemolyticus*	VQTVQIGSD (phage display)	Covalent binding	Sea water Clam extraction Spanish macherel extraction	15	20–1 × 10^4^	210	[85]
Colorimetry	*L. monocytogenes*	Leucocin A (AMP)	Covalent binding		10	10–10^4^	30	[113]
Fluorescence	*E. coli*	Magainin I (AMP)	Covalent binding	PBS	10^3^	10^3^–10^6^	30	[116]
Fluorescence	*E. coli* O157:H7 *S. typhimurium*	Polymyxin B (AMP) Polymyxin E (AMP) Cecropin A (AMP) Magainin I (AMP) Parasin (AMP)	Covalent binding	PBS	1 × 10^5^ 1 × 10^5^	5 × 10^4^–5 × 10^5^ 1 × 10^5^–5 × 10^6^	70	[142]
Fluorescent	*E. coli* O157:H7	Cecropin P1 (AMP) SMAP29 (AMP) PGQ	Covalent binding	PBST 0.05%	10^3^	10^3^–10^7^	30	[143]
Fluorescent	*E. coli* O157:H7 *L. monocytogenes* *B. melitensis*	SLLTPVP (phage display) MWPHPLY (phage display) SGYTRPL (phage display)	Streptavidin-Biotin coupling to MBs	Cabbage	10^3^ 10^2^ 10^2^	10^2^–10^6^	100	[84]
Fluorescence	*S. aureus* *Bacillus*	Protonectin (AMP)	Covalent binding	Peach juice Glucose injection Human urine Lake water	2.2 × 10^2^ 7.3 × 10^2^ 7.8 × 10^2^	2.3 × 10^3^–1.2 × 10^7^	33	[144]
Fluorescence	*L. monocytogenes*	Leucocin A (AMP)	Non-covalent immobilization & covalent binding	Milk	2 × 10^5^	-	50	[145]
Fluorescence	*E. coli* O:146	Indolicidin (AMP)	High concentration zone created with isotachophoresis	Tap water River water	10^4^	10^6^–10^8^	60	[146]
Fluorescence	*E. coli* O157:H7 *E. coli* DH5α	Magainin I (AMP)	Covalent binding	PBS	10	-	20	[147]
Surface plasmon resonance imaging	*S. typhimurium L. monocytogenes* *S. aureus* *S. epidermis* *E. coli* O157:H7	Magainin I (AMP) Clavanin A (AMP) Pediocin (AMP) Leucocin A24 (AMP) PGQ (AMP)	Covalent binding	TSB	6 2.6 × 10^3^ 16 2.5 × 10^3^ 51	-	1080	[111]
Surface plasmon resonance	*E. coli* O157:H7	Magainin I (AMP)	Covalent binding	Drinking water Fruit juice Vegetable juice	5 × 10^2^	10^3^–5 × 10^7^	Real-time	[114]
Electrochemiluminescence	*E. coli*	Magainin I (AMP)	Covalent binding	Drinking water	1.2 × 10^2^	5 × 10^2^–5 × 10^5^		[133]
Microcantilever	*Salmonella* spp. *L. monocytogenes* *E. coli* O157:H7	SEAYKHRQMHMSGGGSC (phage display) NRPDSAQFWLHH (phage display) VPWVTTYEPWGM (phage display) GPADNTSKHVIR (phage display)		PBS	10^6^	10^7^–10^8^	17	[76]
Microcantilever	*L. monocytogenes*	Leucocin A (AMP)	Covalent binding	PBS	10^3^	10^3^–10^6^	Real-time	[135]

## Data Availability

Not applicable.

## Abbreviations

AMP	Antimicrobial peptide
AntiBP	Antibacterial peptide
ATP	Adenosine Triphosphate
BMC	Biomaterial Cantilever
CAMP	Collection of Antimicrobial Peptides
CFU	Colony Forming Unit
CNT	Carbon Nanotube
CPT-1A	Carnitine Palmitoyltransferase 1a
CS	Cysteamine
ECL	Electrochemiluminescence
EFSA	European Food Safety Authority
ELISA	Enzyme-Linked Immunosorbent Assay
EIS	Electrochemical Impedance Spectroscopy
eN	Electronic Nose
FET	Field-Effect Transistors
GE	Gold Electrode
HRP	Horse Radish Peroxidase
LAMP	Loop-Mediated Isothermal Amplification
LOD	Limit Of Detection
LPS	Lipopolysaccharide
LSPR	Localized Surface Plasmon Resonance
MALDI-TOF	Matrix Assisted Laser Desorption Ionization-Time of Flight
MBs	Magnetic Beads
MLT	Melittin
MNP	Magnetic Nano Particle
NCs	Nanoclusters
NHS	N-Hydroxysuccinimide
NIR	Near Infra-Red
NSs	Nanosheets
OBP	Odorant-Binding Protein
ORP	Olfactory Receptor-Derived Peptides
PBS	Phosphate-Buffered Saline
PCR	Polymerase Chain Reaction
PEC	Photoelectrochemical
Ph.D.	Phage Display
PvHS	small subunit of Penaeus vannamei hemocyanin
PWE	Paper Working Electrodes
QCM	Quartz Crystal Microbalance
QDs	Quantum Dots
REASSURED	Real-time connectivity; Ease of specimen collection; Affordability; Sensitivity; Specificity; User-friendliness; Rapid & robust operation; Equipment-free; and Deliverability
RT-PCR	Reverse Transcription Polymerase Chain Reaction
RSD	Relative Standard Deviation
rGo	Reduced Graphene Oxide
SAMs	Self-Assembled Monolayers
SAROTUP	Scanner And Reporter Of Target-Unrelated Peptides
SAW	Surface Acoustic Wave
SELEX	Systematic Evolution of Ligands by Exponential Enrichment
SEM	Scanning Electron Microscopy
SPE	Screen-Printed Electrode
SPR	Surface Plasmon Resonance
SPRI	Surface Plasmon Resonance Imaging
SWCNT	Single Walled-Carbon Nanotube
TMA	Trimethylamine
TMB	tert-Butyl carbamate
TUP	Target-Unrelated Peptide
UCNPs	Upconversion Nanophores
UV	Ultraviolet
VOC	Volatile Organic Compound
WHO	World Health Organization
